# Secretory Lysosome‐Related Gene Signature Defines the Immune Microenvironment and Identifies RGS2 as a Prometastatic Factor in Hepatocellular Carcinoma

**DOI:** 10.1155/humu/3501996

**Published:** 2026-05-16

**Authors:** Zhipeng Ye, BuLang Tang, Yuanjian Zhang, Hanhan Chen, Jie Li, Zhitao Ye, Xuejian Liu, Jiaxi Li, Di Li

**Affiliations:** ^1^ Hepatology Unit, Departments of Infectious Disease, Guangzhou Women and Children’s Medical Center, Guangzhou Medical University, Guangzhou, China, gzfezx.com; ^2^ The Fifth Affiliated Hospital of Guangzhou Medical University, Guangzhou, China, gzhmc.edu.cn; ^3^ Guangzhou Women and Children’s Medical Center, Guangzhou Medical University, Guangzhou, China, gzhmc.edu.cn; ^4^ School of Pharmaceutical Sciences, Guangzhou Medical University, Guangzhou, China, gzhmc.edu.cn; ^5^ Department of Radiology, Xinjiang 474 Hospital, Urumqi, China; ^6^ Department of Clinical Laboratory, Xintang Hospital, Guangzhou, China; ^7^ Department of Ultrasonography, Guangzhou Women and Children’s Medical Center, Guangzhou Medical University, Guangzhou, China, gzfezx.com

**Keywords:** hepatocellular carcinoma, immunotherapy, prognostic signature, RGS2, risk stratification, secretory lysosomes, tumor immune microenvironment

## Abstract

**Introduction:**

Hepatocellular carcinoma (HCC) is a highly aggressive tumour with significant heterogeneity and a poor response to immunotherapy. Secretory lysosomes (SLs) control immunological responses and cellular homeostasis; however, the significance of secretory lysosome‐related genes (SLRGs) in HCC prognosis and treatment is unclear. This work sought to create a predictive signature based on immune lysosome‐related genes (immLysorgs) and to evaluate RGS2’s functional role in HCC progression.

**Methods:**

We identified 13 immLysorgs in MSigDB and evaluated genomic and clinical data from the TCGA‐LIHC and GSE76427 cohorts. Molecular subtypes were identified using nonnegative matrix factorization and consensus clustering. A predictive model (immLysoS) was built using LASSO and multivariate Cox regression with four genes: GZMH, KLRB1, RGS2, and SLC6A1. Model performance was validated across multiple cohorts. The involvement of RGS2 in HCC was examined using functional studies such as CCK‐8, colony formation, migration assays, and a xenograft model.

**Results:**

Two subtypes (C1/C2) and two genotypes (A/B) were found, with the C2 and A groups exhibiting increased survival and immune infiltration. The immLysoS signature efficiently divided patients into risk categories, with low‐risk individuals having much higher overall and progression‐free survival. High immLysoS scores were associated with advanced disease, higher grade, and more TP53 and CTNNB1 mutations. Low‐risk patients responded better to PD‐L1 inhibitors with sorafenib. RGS2 knockdown drastically reduced HCC cell proliferation, migration, and tumor development both in vitro and in vivo.

**Discussion:**

The immLysoS signature is an effective tool for risk assessment and therapy advice in HCC, as it links SL‐related genes to immunological characteristics and therapeutic response. RGS2 emerges as a possible oncogenic driver and therapeutic target, necessitating additional research into its processes in the HCC microenvironment.

## 1. Introduction

Hepatocellular carcinoma (HCC) is a prevalent malignancy with a high death rate. Global tumor epidemiological data from 2010 to 2019 show that HCC ranked fifth in global cancer incidence and third among the most lethal tumors [1]. The 5‐year survival rate for HCC patients is about 14.1% [2], primarily due to advanced disease at clinical presentation, limited treatment options, and poor prognosis factors including high heterogeneity, metastasis, impaired liver function, and overall physical deterioration [3–5]. There has been a rising awareness of the significance of the tumor immune microenvironment (TIME) in tumor development [6, 7]. Immunotherapy with immune checkpoint inhibitors (ICIs) has become a very promising treatment method for several malignancies since it can effectively restore immune cell activity and destroy tumors [8–10]. Although immunotherapy is also becoming common for systemic HCC treatment, it has yet to achieve high response rates in clinical practice [11, 12]. The tumor microenvironment (TME) in HCC is complicated because several immune and stromal cells work together to generate an immunosuppressive milieu that compromises the efficacy of immunotherapies in HCC [8]. Thus, elucidating the genetic landscape of HCC and establishing robust prognostic indicators are imperative for advancing existing treatment regimens and improving patient survival outcomes.

Lysosomes are membrane‐enclosed cytoplasmic organelles packed with acid hydrolases, featuring an acidic lumen and a lysosomal membrane composed of a phospholipid bilayer. Under physiological conditions, lysosomes are involved in crucial activities such as biomolecule degradation, innate and adaptive immune responses, and nutrient sensing [13]. Research indicates that lysosomes may facilitate the progression and invasive metastasis of malignant tumors by modulating tumor cell proliferation, invasion, and resistance to radiation mechanisms [14]. Secretory lysosomes (SLs) are a specialized subtype of lysosomes that perform conventional lysosomal tasks, including the breakdown of aged proteins, while additionally acting as a reservoir for newly synthesized secretory proteins [15]. SLs are most abundantly expressed in immune and specialized cells, and their primary biological role is to regulate immune responses and maintain immune homeostasis [16, 17].

Dynamic modulation of secretory lysosomal signaling cascades may enhance the cytotoxic activity of lymphocytes, which could be useful in cancer immunotherapy [18]. Over the past 5 years, a growing number of lysosome‐associated gene signatures have been used to predict outcomes of cancer patients [19–23]. Nonetheless, there is an urgent need for more reliable and precise markers to forecast immunotherapy response and survival in patients with HCC. Moreover, the precise regulatory functions of secretory lysosome‐related genes (SLRGs) in influencing immunotherapy effectiveness and HCC prognosis are predominantly uninvestigated.

In this context, we performed a comprehensive analysis based on SLRGs. We developed a novel HCC prognostic marker, immLysoS, and validated it thoroughly to predict HCC patients’ prognoses and the effectiveness of immunotherapy. Notably, in vitro experiments demonstrated that RGS2 may inhibit HCC progression, which gives us a good starting point for creating lysosome‐targeted therapies for HCC.

## 2. Materials and Methods

### 2.1. Data Collection and Cleaning

Thirteen immune lysosome‐related genes (immLysorgs) were extracted from the “GOCC_CYTOLYTIC_GRANULE” gene set in MsigDB. SLs are a class of specialized lysosomes with cytotoxic granule functions. In immune cells, they mainly exist in the form of cytotoxic granules, which are responsible for storing and releasing effector molecules such as perforin and granzymes. The “GOCC_CYTOLYTIC_GRANULE” gene set in the MSigDB database is currently the most authoritative collection of cytotoxic granule‐related genes. The genes included in this set have all been confirmed to be involved in the formation, transport, and secretion of cytotoxic granules, and their functions highly overlap with those of SLs. Therefore, screening immLysorgs from this gene set has a sufficient theoretical basis. The GDC database was additionally queried to acquire the TCGA‐LIHC cohort data with 371 tumor samples and 50 normal samples. One hundred and thirty‐five liver cancer samples were chosen from this group because they had detailed expression profiles and information on clinical survival. One hundred and sixty‐seven samples from the GSE76427 dataset were used as an external validation set. ICI anticipated response scores were obtained from the TCIA database, and the GDC database was queried for variant nucleotide data in mutation annotation format (MAF). We also retrieved the TCGA‐LIHC cohort’s copy number variation (CNV) data from UCSC Xena.

### 2.2. Genetics and Prognostic Landscape Development for immLysorgs

The “limma” R package was used to do a differential study of samples of HCC and paraneoplastic samples. The TCGA‐LIHC samples were split into groups with high and low expression levels using the best cutoff value for the gene expression profile to see how it related to prognosis. The “log‐rank” and “one‐way Cox regression” methods were used to compare the overall survival (OS) rates of these groups. Then, a gene predictive coexpression network was set up to show the Pearson relationship between the 13 immLysorgs and the risk ratio (HR) found by univariate Cox regression.

### 2.3. Initial Nonnegative Matrix Factorization (NMF) Clustering Predicated on immLysorgs

We used NMF to group the expression patterns of the 13 immLysorgs from the TCGA‐LIHC dataset [24]. After 10 iterations of the Brunet method, the optimal categorization number was found to be the one that showed the fastest cophenetic fall. After this, different molecular subtypes were assigned to each sample. The expression distribution of the 13 immLysorgs across different subtypes was visualized and downscaled using principal component analysis (PCA). Disparities in OS across patients with different subtypes were subsequently evaluated using Kaplan–Meier (KM) survival analysis.

### 2.4. Gene Set Variation Analysis (GSVA)

To explore the potential variability in biological process (BP) across different subtypes, GSVA was conducted on the gene set “c2.cp.kegg.v7.5.1.symbols.gmt.”

#### 2.4.1. Analysis of Differences in Immune Microenvironment Between immLysorg Clusters

Infiltration levels of immune cells within the TIME were quantified using the single‐sample gene set enrichment analysis (ssGSEA). The “ESTIMATE” R software was used within the analysis. ESTIMATEScore shows the relative abundance of stromal and immunological elements, which is the sum of StromalScore and ImmuneScore.

### 2.5. Analysis of Variance and Enrichment Analysis

We used the “limma” R package to do a differential analysis of numerous immLysorg groups in order to uncover differentially expressed genes (DEGs). We used the “clusterProfiler” R package to do functional enrichment analysis on the DEGs to find out what biological activities and pathways.

### 2.6. Screening Genes Based on Subtype Using Random Forest (RF)

The RF approach was used to identify genes specific to subtypes, where the model was deemed strong after 500 decision trees were created, with the default iteration number set to 100. The genes that were chosen for the subtype‐specific artificial neural network (NN) model were those with a score higher than 4.

### 2.7. Artificial NN Construction of Subtype Differentiation Model

The subtype feature genes were used to train an artificial NN model using the “neuralnet” and “NeuralNetTools” R packages. Then, the genes were given weight information and scores using the “0/1” assignment approach. The “pROC” R package was used to output the model’s subtype prediction and evaluate its classification ability.

### 2.8. Unsupervised Clustering Analysis

We identified differentially expressed prognostic genes (DEPGs) by subjecting the DEGs to one‐way Cox regression analysis. In a second round of unsupervised clustering, the “ConsensusClusterPlus” R software was employed with the DEPG expression profiles. Various genetic subgroups were identified from the samples in this way. Patients with various genotypes were compared using the KM survival analysis to determine prognostic differences.

### 2.9. Construction and Validation of immLysoS

The following phase of the least absolute shrinkage and selection operator (LASSO) regression was used to select protein‐encoding genes from the group of DEPGs. Finally, the modeled genes were tested, and the immLysoS was built using multivariate Cox regression. “Immune lysosome possibly related genes” (ILPRGs) were the names given to the mimicked genes. To locate immLysoS, we followed these steps: Let *β* represent the regression coefficient and h0(*t*) the baseline risk function. ImmLysoS is equal to h0(*t*) times exp(*β*_1_ *X*_1_ + *β*_2_ *X*2+⋯+*β*_n_ *X*_*n*_). Two groups were established in the TCGA‐LIHC training set for patients with liver cancer: One group had a high median immLysoS value, and the other had a low median immLysoS value. Sankey plots were then employed to illustrate the correlation between immLysorgcluster, gene cluster, immLysoS grouping, and the persistence of liver cancer cases within the TCGA cohort. After this, we employed analysis of variance to assess the differences in immLysoS between the gene cluster and the immLysorgcluster. In addition, we examined the impact of progression‐free survival (PFS) on OS prediction and used KM survival analysis to compare OS changes between the high and low immLysoS groups in the TCGA training set, internal validation set, and external validation set. The accuracy of the OS prediction of immLysoS at 1, 3, and 5 years was then evaluated.

### 2.10. Clinical Subgroup Analysis

The proportional distribution of clinical features across subgroups was quantified, compared, and visualized using bar charts to contrast the high and low immLysoS groups. The clinical subgroups “Stage” and “Grade” were thought to be significant for HCC patients, and variations in immLysoS among patient subgroups with varying stages and grades were compared using a differential analysis. The KM survival analysis was also applied to examine the effect of immLysoS grouping on OS in these patient sets.

### 2.11. Mutation and Stemness Analysis Based on immLysoS

To create distinct mutational landscapes for high and low immLysoS groups, Maftools software was employed. Corresponding TMB values were determined from the liver cancer samples in each TCGA cohort. We performed differential and correlation analyses to compare TMB levels between high and low immLysoS groups and assess their association with immLysoS. Furthermore, the association between immLysoS and tumor cell stemness, represented by a tumor stemness score (RNAs) derived from mRNA expression patterns [25], was also examined via correlation analysis.

### 2.12. Immune Microenvironment and Immunotherapy Efficacy Analysis

The immune infiltration landscape in the TIME was constructed using the ssGSEA and ESTIMATE algorithm. Correlation matrices were constructed between 23 immune cells and immLysoS. Additionally, correlation matrices were also used to show that immLysoS may be associated with 46 immunological checkpoints. The predictive value of immLysoS in the PD‐L1 monoclonal antibody treatment group was assessed using the IMvigor210 immunotherapy cohort and KM analysis.

### 2.13. Sorafenib Sensitivity Analysis

We calculated the expected half‐inhibitory concentration (IC50) of sorafenib utilizing the drug data from the Genomics of Drug Sensitivity in Cancer (GDSC) and the outcomes from ridge regression of the calcPhenotype function applied to gene expression profile data. We next used analysis of variance to compare the differences in IC50 estimations of sorafenib between different immLysoS groups.

### 2.14. Cell Culture

The human normal hepatocytes (LX2) and HCC cells (Hep3B and MHCC97H) were acquired from the Shanghai Cell Bank of the Chinese Academy of Sciences. Using Lipofectamine 2000, stable RGS2‐knockdown cell lines were established. The pLKO.1‐puro vector served as the basis for all three plasmids. The sequences 5 ^′^‐GACCCGTTTGAGCTACTTCTT‐3 ^′^ and CGGAGAAATCTATTGAAGCAT were used as targets for shRGS2#1 and shRGS2#2, respectively.

### 2.15. Reverse Transcription Quantitative Real‐Time PCR (RT‐qPCR)

RNA was extracted using fast reagent (Invitrogen). If the A260/A280 ratio was more than 1.8, spectrophotometry was used to confirm that the RNA was pure. We performed reverse transcription of 1 *μ*g of total RNA with a Reagent Kit from TaKaRa (Shiga, Japan) to generate cDNA. The 2^−*Δ* *Δ*Ct^ technique was used to quantify relative RNA expression after normalization with GAPDH as an internal reference. Primers utilized in this investigation were as follows:

H‐GZMH: F: 5 ^′^‐CTGGCTGGGGTTATGTCTCAA‐3 ^′^, R: 5 ^′^‐GGCTACGTCCTTACACACGAG‐3 ^′^.

H‐KLRB1: F: 5 ^′^‐CCCTTGGAATAACAGTCTAGCTG‐3 ^′^, R: 5 ^′^‐TTGTCACGTATCAGGTTCTGTG‐3 ^′^.

H‐RGS2: F: 5 ^′^‐AAGATTGGAAGACCCGTTTGAG‐3 ^′^, R: 5 ^′^‐GCAAGACCATATTTGCTGGCT‐3 ^′^.

H‐SLC6A1: F: 5 ^′^‐GGGTATGGAAGCTGGCTCCTA‐3 ^′^, and R: 5 ^′^‐AGGGGTTGTCGCACTGTTTC‐3 ^′^.

### 2.16. Western Blotting

To extract total protein, cells collected in the logarithmic growth phase were lysed with RIPA lysis solution (PC101, Epizyme). Next, proteins were transferred onto PVDF membranes after electrophoresis. After blocking using Rapid Blocking Buffer (PS108, Epizyme), the membranes were treated with a primary anti‐RGS2 antibody (10678‐1‐AP, diluted 1:1000, Proteintech, China). Following three washes with TBST, the membranes were incubated for 60 min at 37°C with a horseradish peroxidase (HRP)–conjugated goat anti‐rabbit IgG (H + L) secondary antibody (AWS0002, 1:5000 dilution, Abiowell, China). The protein bands were then examined with imaging software (SCG‐W3000 PLUS, Servicebio, China) and Omni‐ECLTM Ultra‐Sensitive Chemiluminescent Substrate (SQ201, Epizyme, China).

### 2.17. Cell Viability and Proliferation Assay

A Cell Counting Kit‐8 (CCK‐8, Biosharp, BS350B) was used to check the viability of the cells. In each well of a 96‐well plate, 2000 cells were seeded in 100 *μ*L of complete media; cells were exposed to various concentrations of sorafenib for 48 h. Ten microliters of CCK‐8 solution was added to each well according to the instructions of the kit. A BIOTEK ELX800 microplate reader then logged the absorbance at 450 nm. Over the course of 5 days, this test was run in a straight line.

### 2.18. Colony Formation Assay

Eight hundred cells were distributed evenly throughout the six wells of the plate, and the medium was changed regularly while colony formation was being observed. When the number of cells in one colony reached 50, as seen under a microscope, the culture was halted. Then, they were fixed and stained by incubating with 1 mL of crystal violet solution per well for 30 min. Following multiple washes with PBS, pictures were taken, and colonies were tallied using ImageJ software.

### 2.19. Wound Healing and Cell Migration Assay

Cells transfected with Hep3B and MHCC97H were incubated at 37°C. Upon attaining 70%–90% confluence, a 200 *μ*L pipette tip was employed to trace a perpendicular line across the base of the well, thereby creating a monolayer scratch. Two washes with PBS were then used to remove the cells that had detached. At 0 and 24 h, the scratch was photographed under a 4× microscope, and its breadth and exact location were noted.

As for the cell migration assay, we put 1 × 10^5^ transfected cells in serum‐free medium into the upper chamber, and put the upper chamber into a plate of medium with 10% FBS in the lower chamber. Using a cotton swab, we took out the cells that had not moved from the upper chamber after they had been in the incubator at 37°C for a night. The last stage was to use pictures taken under a microscope to count the cells in three random areas.

### 2.20. Xenograft Tumor Model

This study complied with all relevant ethical regulations. All animal procedures were approved by the Animal Welfare and Ethics Committee. Five‐week‐old female BALB/c nude mice were purchased and housed in the SPF‐grade Animal Experimental Center. After 1 week of adaptive feeding, mice were randomly divided into four groups (*n* = 5 per group). Hep3B cells stably transfected with shNC or shRGS2#1 (2 × 10^6^ cells resuspended in 200 *μ*L PBS) were subcutaneously injected into the left posterior flank of each mouse. Tumor dimensions were measured every 3 days using a vernier caliper, and tumor volume was calculated with the following formula: *V* = (length × width^2^)/2. At the experimental endpoint, mice were euthanized, and tumor tissues were harvested for weighing, imaging, and histological analysis.

### 2.21. Statistical Analysis

R (Version 4.2.1) and Perl (mostly for batch cleaning of data) were used to perform bioinformatics analysis. Unless otherwise noted, the “limma” R package was employed for variance analysis. The Wilcoxon rank‐sum test was used for comparison of nonparametric data between two groups, and the Kruskal–Wallis *H* test was used for comparison of nonparametric data among three or more groups. Statistical significance was defined as a two‐tailed *p* value < 0.05.

## 3. Results

### 3.1. Differential Expression, Genetic Alterations, and Prognostic Significance of immLysorgs

Initially, a set of 13 immLysorgs (GNLY, SERPINB1, GZMH, GZMB, LAMP1, NKG7, ARL8B, PRF1, SRGN, STXBP2, TIAL1, CALR, and RNF19B) was obtained from the MsigDB database. Table 1 summarizes the basic information of the 371 liver cancer samples that were included in the analysis cohort from the TCGA database. In order to differentiate between cancerous and noncancerous tissues, the TCGA database was utilized to conduct immLysorg differential expression analysis. As shown in Figure S1A, NKG7, PRF1, and SRGN showed considerably higher expression in normal tissues. In contrast, the expression of RNF19B, SERPINB, LAMP1, ARL8B, STXBP2, TIAL1, and CALR in tumor tissues was significantly upregulated.

**Table 1:** Baseline data sheet for the cohort of TCGA‐LIHC.

Characteristic	Levels	*N*(%)
Age	≤ 65 years old	230 (62.0%)
	> 65 years old	141 (38.0%)

Gender	Female	120 (32.3%)
	Male	251 (67.7%)

Stage	I	174 (50.1%)
	II	85 (24.5%)
	III	84 (24.2%)
	IV	4 (1.2%)

Grade	G1	55 (15.0%)
	G2	178 (48.6%)
	G3	120 (32.8%)
	G4	13 (3.6%)

AFP	≤ 400 ng/mL	219 (77.7%)
	> 400 ng/mL	63 (22.3%)

immLysorgs’ mutation profile and CNV frequency were next examined. Figure S1B shows that out of 371 HCC samples, 15 (4.04%) had somatic cell mutations, with CALR (1%) and PRF1 (1%) in particular exhibiting high mutation frequencies. Intriguingly, whereas RNF19B, CALR, TIAL1, and STXBP2 exhibited substantially higher copy number deletion frequencies compared to increased frequencies, the frequency of copy number increases for LAMP1, SERPINB1, GZMH, and GZMB was noticeably greater than the deletion frequency. Specifically, as seen in Figure S1C, NKG7 only exhibited variations involving copy number deletions. Figure S1D also shows that copy number variants of various genes were found throughout the genome. Specifically, copy number variants of NKG7, CALR, and STXBP2 were found on Chromosome 19, GZMB and GZMH on Chromosome 14, and TIAL1, PRF1, and SRGN on Chromosome 10, and other genes were scattered throughout the rest of the genome.

Univariate Cox regression analysis was subsequently applied to investigate the predictive relevance of immLysorgs in HCC patients. Figure S2A–L shows that, with the exception of GNLY, all 12 of the remaining genes in immLysorgs were strongly linked to OS. Protective genes in HCC patients were found to be GZMB, GZMH, NKG7, PRF1, and SRGN, according to prognostic network plots, which showed a strong positive connection. However, as shown in Figure S2M, the remaining eight genes in immLysorgs were identified as risk genes in patients with HCC.

### 3.2. Molecular Typing Based on NMF With GSVA, ssGSEA, and ESTIMATE Analysis

The expression levels of immLysorgs were used to perform molecular typing using NMF. Categorization of HCC patients into two major subgroups, C1 and C2, was achieved by selecting the ideal rank value (Figure 1A,B). There was a definite separation of the two categories in the PCA data (Figure 1C). Among the immLysorgs, for example, there was a notable difference in the expression of GNLY, RNF19B, GZMH, GZMB, NKG7, PRF1, SRGN, and STXBP2, and with the exception of LAMP1, all of these genes showed elevated expression levels in the C2 subgroup (Figure 1D).

Figure 1Identification of immune lysosome–related subtypes and exploration of their clinical and biological features. (A, B) Nonnegative matrix factorization clustering dividing HCC samples into two clusters (*k* = 2) based on 13 immLysorgs. (C) PCA of the two isoforms. (D) Differential expression of immLysorgs between the two subtypes. (E) OS curves for the two subtypes of patients with HCC. (F) Different distribution of clinicopathological features and immLysorg expression among the two subtypes. immLysorgs; immune lysosome‐related genes;  ^∗^ *p* < 0.05;  ^∗∗^ *p* < 0.01;  ^∗∗∗^ *p* < 0.001.

**Figure:**
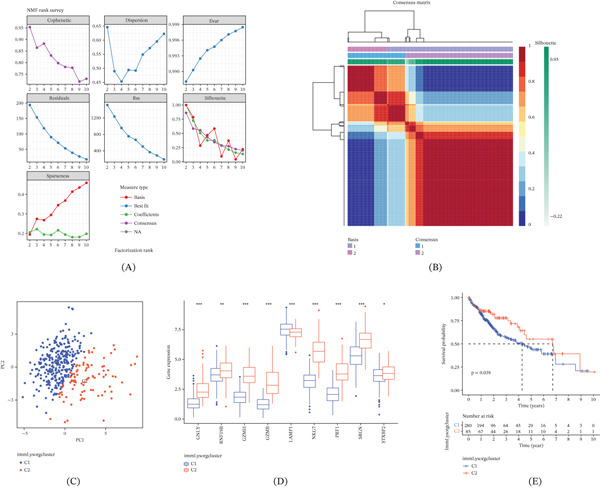
(A)

**Figure:**
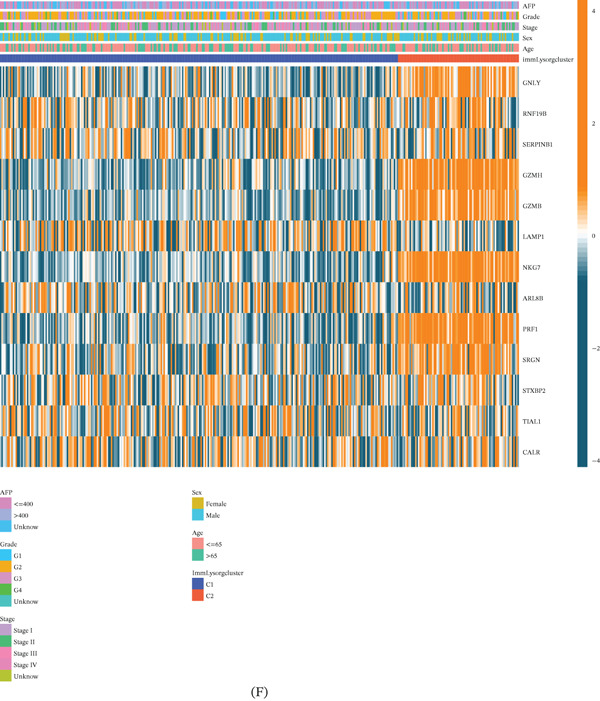
(B)

**Figure 2:**
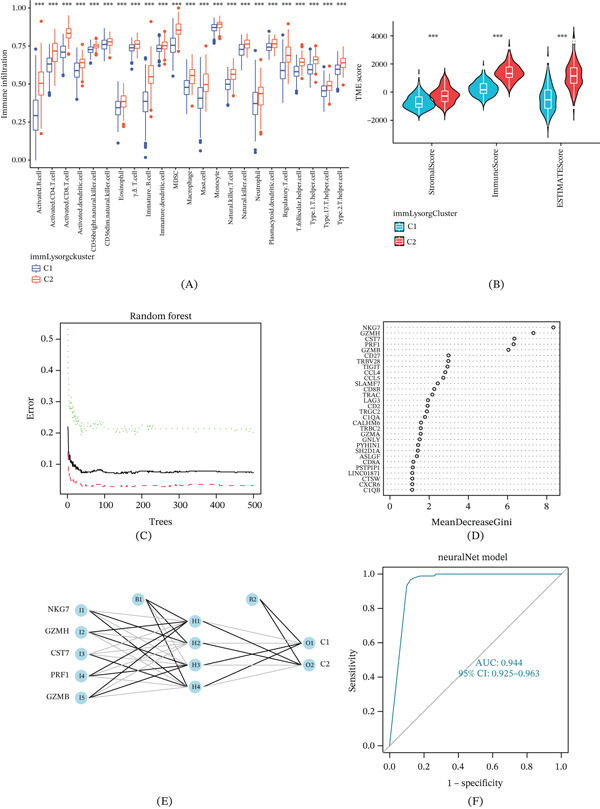
Analysis of differences in immune cell infiltration, TME scores, and random forest algorithm between the subtypes. (A) Differential analysis between immune cells and the scale of fraction for Cluster C1 and Cluster C2. (B) Differential analysis between the TME score for Cluster C1 and Cluster C2. (C, D) Random forest algorithm analysis of 417 differential genes between C1 and C2 subtypes. (E) Construction of an ANN model for subtype classification. (F) ROC curve of the ANN model.  ^∗∗∗^ *p* < 0.001.

A statistically significant difference in OS was found between the subgroups based on KM analysis (Figure 1E). Patients in C2 had the best prognosis. The corresponding heat map generated from NMF clustering delineates the clinical characteristics—including age, gender, stage, grade, and AFP level—across these distinct HCC patient subgroups from TCGA (Figure 1F). Subsequently, each subgroup underwent GSVA. These findings showed that the C2 subgroup exhibited upregulation of most signaling pathways, especially those associated with the immune system (Figure S3). Figure 2A shows that compared to the C1, the C2 subgroup had considerably higher infiltration levels of 23 immune cells.

Finally, Figure 2B shows that compared to the C1 subgroup, the C2 subgroup had considerably higher values for ImmuneScore, StromalScore, and ESTIMATEScore. These findings point to a stronger correlation between immunological‐related pathways, levels of immune cell infiltration, and ESTIMATEScore, which in turn suggests that patients in the C2 category have a more favorable clinical prognosis. Immunotherapy may thus work much better for people in the C2 subgroup.

### 3.3. Enrichment Analysis of DEGs and RF Model Construction

We performed a differential study of gene expression patterns by comparing individuals in C1 and C2 subgroups. Using a volcano plot (Figure S4A), we were able to visualize 417 DEGs that were detected by applying the criterion of logFoldChange (logFC) > 1 and FDR < 0.05. To understand the possible roles and pathways linked to the DEGs, we ran KEGG and GO enrichment studies.

According to the GO study of the BP category, the DEGs mostly function in cellular immune processes, intracellular immune responses, and immunological signaling. The DEGs belonged to the cellular component (CC) class and were shown to be tightly linked to immunoglobulin complexes situated on the plasma membrane’s outer surface. As shown in Figure S4B,D, the DEGs may also affect antigen binding and immunoglobulin receptor binding. DEGs may also be involved in multiple pathways, as suggested by KEGG enrichment analysis. These pathways include interactions between cytokines and their receptors, cell adhesion molecules, hematopoietic cell synthesis, differentiation of T helper 17 cells, infection with *Staphylococcus aureus*, and so on. Figure S4C,E further shows that the DEGs were linked to pathways that process and present antigens and chemokine pathways. Using an RF model and classification on the 417 differential genes, we were able to identify critical genes for the C1 and C2 subgroup types. The Top 5 most significant genes were NKG7, GZMH, CST7, PRF1, and GZMB. Based on the correlation plot of RF branch number and model error, the optimal number of decision trees for the final model was determined and set at 500 (Figure 2C,D).

In addition, we built a NN model. Ten layers made up the NN: five for input, four hidden, and two for output (Figure 2E). Figure 2F shows the ROC curves representing the cross‐validation findings; they show that the model built using the five genes was quite dependable, with an AUC value of 0.944 (0.925–0.963). Based on these results, the five genes are found to be very important for subgroup identification (C1 and C2).

### 3.4. Consensus Clustering Typing and Prognostic Model Building Based on DEPGs

**Figure 4:**
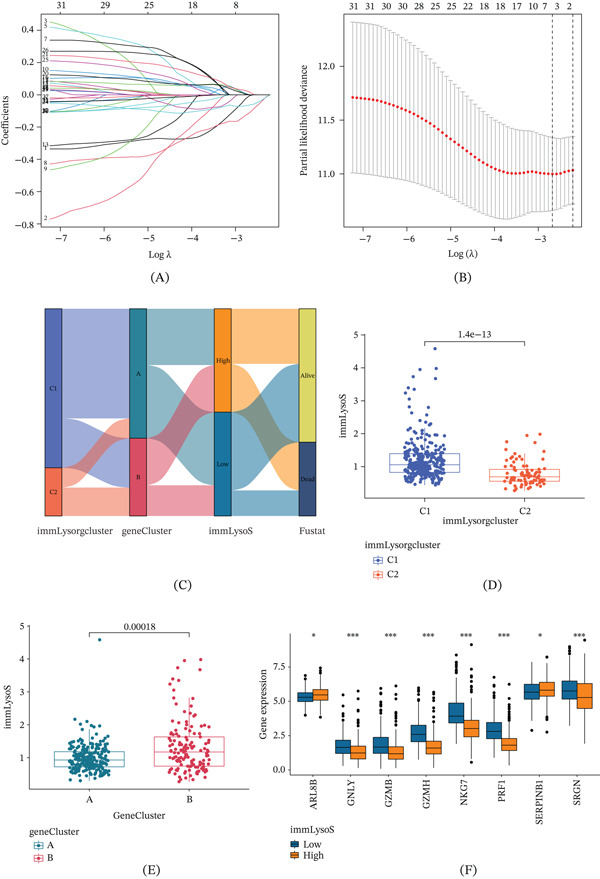
Signature clustering analysis. (A, B) LASSO regression analysis and multivariate Cox for screening SigGenes. (C) ggalluvial shows the construction of the prognostic model. (D) Boxplots indicate the differences in risk scores in the immLysorg cluster and (E) gene cluster. (F) Differential analysis of immLysorgs expression. SigGenes, significant genes; immLysorgs, immune lysosome‐related genes;  ^∗^ *p* < 0.05;  ^∗∗^ *p* < 0.01;  ^∗∗∗^ *p* < 0.001.

Next, we examined the 417 DEGs using one‐way Cox analysis and found 39 DEPGs. Figure 3A,B shows the results of our use of consensus matrices and delta areas to divide HCC patients into Genetic Subtypes A and B, respectively, in order to build a new lysosome score system based on unsupervised consensus clustering using DEPGs. Patients in Group A had a far better OS and PFS (Figure 3C,D) than those in Group B, and in Group A, the expression of 12 genes was noticeably reduced, with the exception of LAMP1 (Figure 3E). Further, we combined age, gender, stage, grade, and AFP level of HCC patients from TCGA to create a ConsensusCluster‐based heat map (Figure 3F). Patients in Group A may have had a better prognosis since their tumors were staged earlier and their histological grades were lower, according to the study. After that, we used a 3:2 ratio to split the TCGA HCC patients into two groups: one for training (*n* = 221) and another for internal validation (*n* = 143). We screened 32 protein‐coding genes from the DEPGs in the training group using LASSO and multivariate Cox regression (Figure 4A,B) before determining that the four best genes for building a risk score formula were GZMH, KLRB1, RGS2, and SLC6A1.

Figure 3Identification of two gene subtypes and exploration of their clinical and biological features. (A, B) Unsupervised consensus clustering dividing HCC samples into two gene clusters based on 39 DEPGs (*k* = 2). (C) OS curves for the two gene subtypes of patients with HCC. (D) PFS curves for the two gene subtypes of patients with HCC. (E) Expression difference analysis of 13 significant immLysorgs between the two gene subtypes. (F) Distribution of differences in clinicopathological features between the two clusters and two gene subtypes. immLysorgs, immune lysosome‐related genes; DEPGs, differentially expressed prognostic genes with strong prognostic significance;  ^∗^ *p* < 0.05;  ^∗∗^ *p* < 0.01;  ^∗∗∗^ *p* < 0.001.

**Figure:**
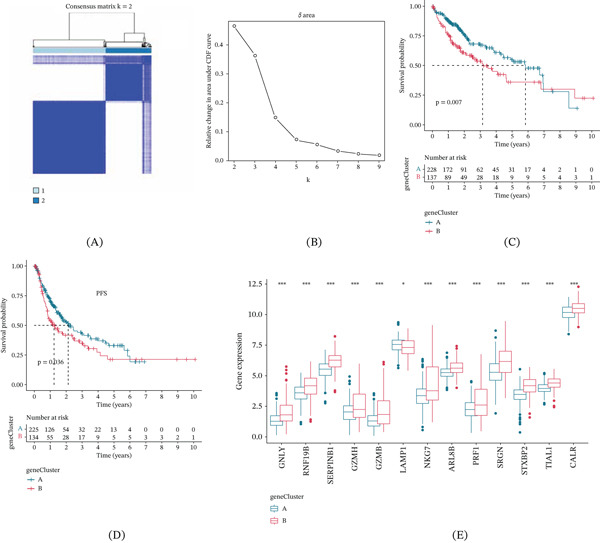
(A)

**Figure:**
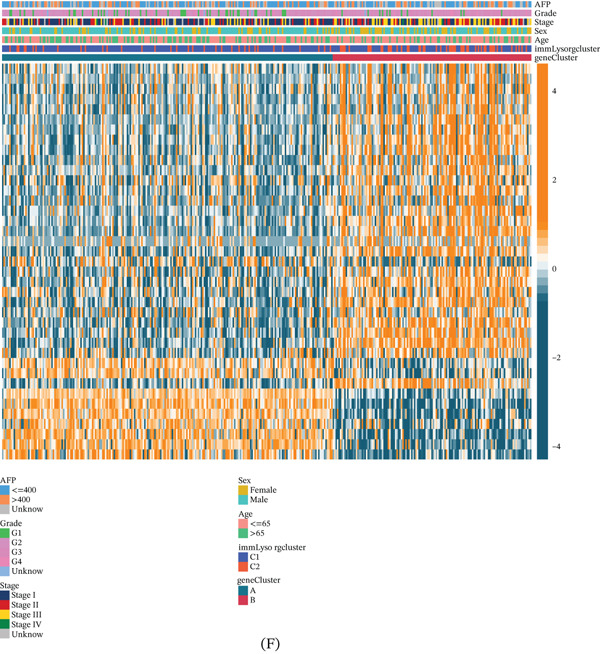
(B)

These four genes were therefore used to establish immLysoS using the following formula: immLysoS = exp (GZMH × (−0.195)) + exp (KLRB1 × (−0.265)) + exp (RGS2 × 0.148) + exp (SLC6A1 × (−0.117)). Using the median as the dividing point, patients were subsequently classified into either the low‐risk or high‐risk group. In Figure 4C, the Sankey diagram shows how the prognostic model was built and how the immLysorgcluster, gene cluster, and immLysoS grouping corresponded to survival. In addition, when comparing immLysoS scores across various subtypes of ImmLysorgsCluster (C1 and C2) and gene cluster (A and B), we found that Groups C2 and A had markedly lower immLysoS scores than Groups C1 and B, respectively (Figure 4D,E).

A differential expression study of 13 immLysorgs genes, ARL8B, GNLY, GZMB, GZMH, NKG7, PRF1, SERPINB1, and SRGN, showed statistically significant variations between these 2 groups using immLysoS (Figure 4F). The results above show that immLysoS, which is built on immLysorgs, can serve as a reliable predictor of HCC patient prognosis with potential clinical utility.

### 3.5. Validation of the Predictive Value of ImmLysoS

**Figure 5:**
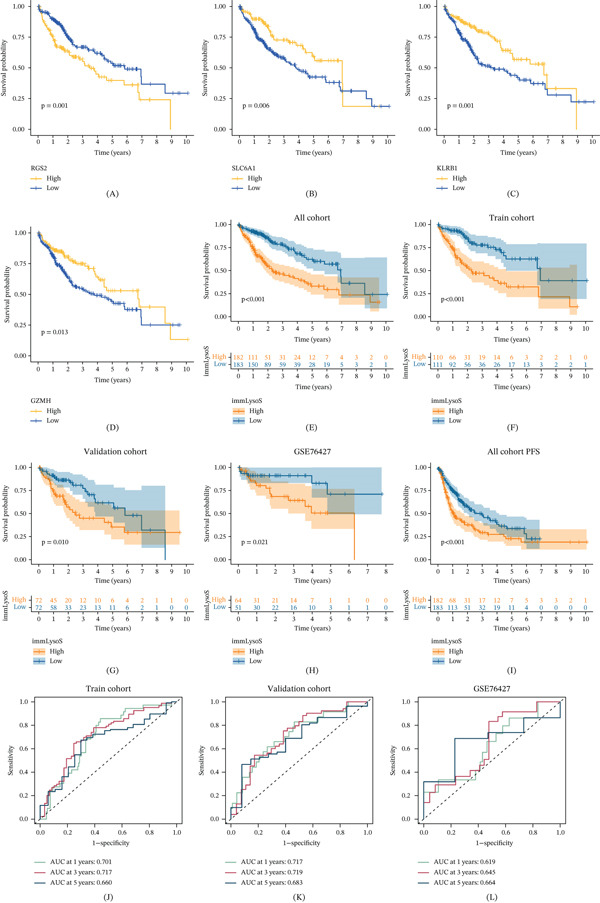
Validation of the prognostic value of signatures. (A–D) The OS curve of four SigGenes of patients with HCC. (E–H) The OS curve of all sets, the training set, the validation set, and the GSE76427 set. (I) The PFS curve of all sets. (J–L) ROCs for 1‐year, 3‐year, and 5‐year OS prediction in the training, validation, and GSE76427 sets. SigGenes, significant genes.

KM analysis revealed significant correlations between the expression levels of RGS2, SLC6A1, KLRB1, and GZMH and the prognosis of HCC patients (Figure 5A–D). Furthermore, we compared the high‐ and low‐risk groups in training, internal validation, and external validation cohorts to determine whether immLysoS differed in terms of prognosis (Figure 5E–H). Using the immLysoS gene signature to generate risk scores, KM curves showed that HCC patients’ OS was considerably different, with the high‐risk group experiencing worse OS. Furthermore, as shown in Figure 5I, immLysoS showed robust predictive accuracy for patient PFS.

Besides, we also plotted ROC curves at 1‐, 3‐, and 5‐year intervals for training, internal validation, and external validation cohorts (AUC > 0.619 for all time periods, Figure 5J–L) and examined the distribution of immLysoS‐based groups in different clinical categories of HCC patients. Patients of diverse ages, sexes, and AFP expression levels showed no significant differences in the distribution of scores between high‐ and low‐risk groups (Figure 6A–C), but when looking at patients with varying stages and histologic grades, the scores from different groups were linked to more advanced stages and histologic grades (Figure 6D,E). Patients with Stage I and Stage III, as well as Grade 1 and Grade 3, showed the most noticeable changes in the distribution discrepancies of immLysoS when compared to other stages and histological grades. Despite these variations, immLysoS was still able to predict OS in patients with low‐grade and high‐stage liver cancer, as well as those in the early and late stages (Figure 6F–K). All things considered, our results show that immLysoS could be useful for predicting how HCC patients may fare in the future.

Figure 6The relationship between five clinical subgroup characteristics and immLysoS using proportional distribution, analysis of variance, and KM curve analysis. *p* values for analysis of variance between two groups are shown on the horizontal line. Proportional distribution of five clinical subgroup characteristics and immLysoS: (A–E) age, sex, AFP, stage, grade (histological grade). (F–H) Distribution of differences, G1–G2 and G3–G4. (I–K) Distribution of differences, Stages I–II and Stages III–IV. ImmLysoS, immune lysosome score.

**Figure:**
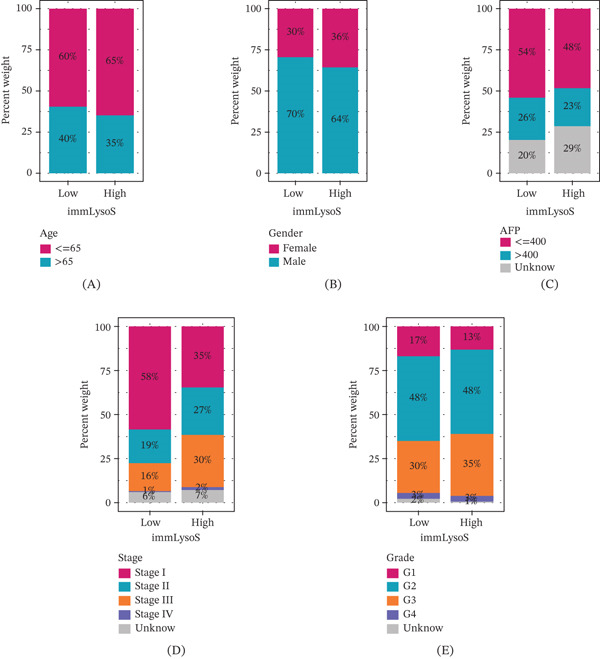
(A)

**Figure:**
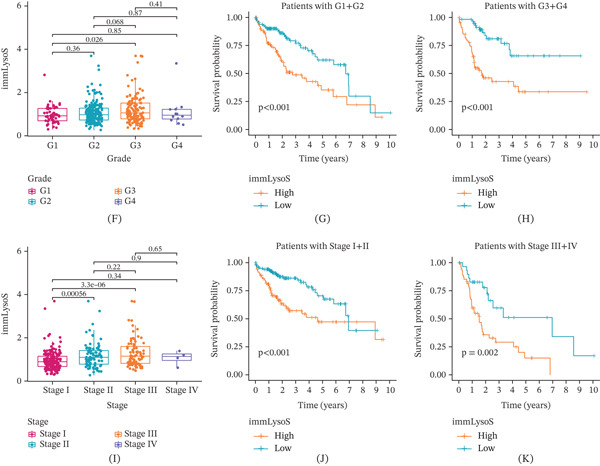
(B)

### 3.6. Correlation of ImmLysoS With TME, RNA Expression, Gene Mutation Frequency, and TMB

We examined the correlation between immLysoS scores and the TME. Figure 7A shows that immLysoS scores were inversely associated with ImmuneScore, StromalScore, and ESTIMATEScore. We further examined the relationship between immLysoS scores and RNA expression levels and observed a statistically significant positive correlation (Figure 7B). Similarly, we examined the gene mutation rates across various immLysoS groups. The findings showed that the group with a high immLysoS score was more likely to have mutations in TP53, CTNNB1, TTN, LRP1B, and OBSCN, all of which are typically linked to a poor prognosis in HCC (Figure 7C,D). Finally, we investigated the potential association between immLysoS scores and TMB in HCC patients but observed no significant correlation (Figure 7E,F). Based on these findings, it seemed that immLysoS’s prognostic guidance in HCC was not based on TMB but rather on the TME, RNA expression, and gene mutation frequency.

Figure 7Assessment of tumor microenvironment, gene mutation frequency, and TMB in different immLysoS groups. (A) Comparison of interstitial scores, immune scores, and estimated scores between high immLysoS and low immLysoS groups. (B) Correlation between RNAs and immLysoS. (C, D) Comparison of gene mutation frequencies between high immLysoS and low immLysoS groups. (E, F) The association between tumor mutational load and different immLysoS groups. ImmLysoS, immune lysosome score;  ^∗∗∗^ *p* < 0.001.

**Figure:**
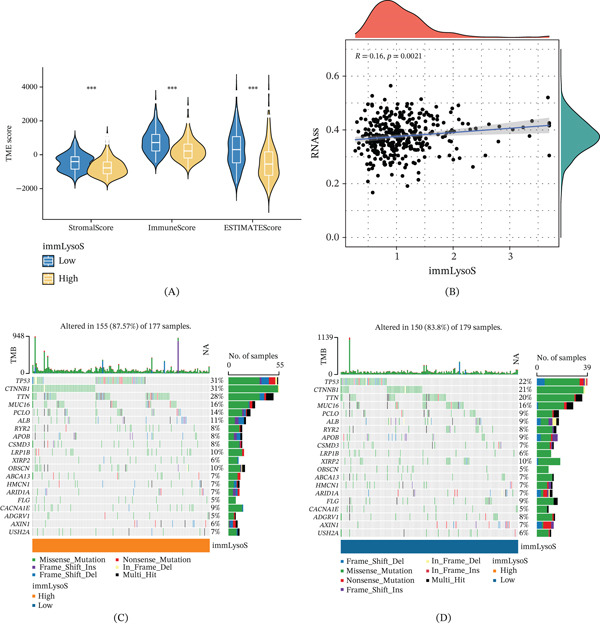
(A)

**Figure:**
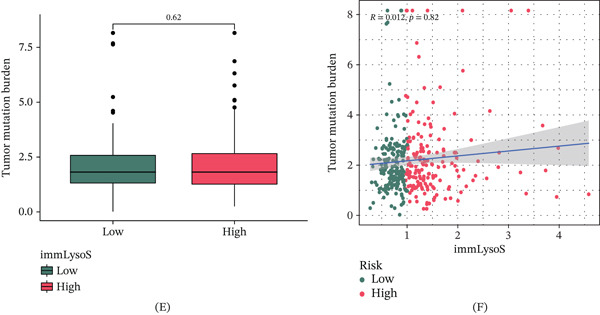
(B)

### 3.7. Gene Set Enrichment Analysis With ImmLysoS

By comparing the gene sets of HCC patients stratified by immLysoS levels, we were able to obtain a better understanding of the possible BPs and pathways linked to the immLysoS‐based differential prognosis. There was a notable enrichment of various Reactome collection gene sets in the high‐risk group. These sets included genes involved in cell cycle checkpoints, histone deacetylation, M phase, and mitotic prometaphase. Among the low‐risk individuals, there were enriched gene sets linked to anti‐inflammatory responses that promoted leishmanial parasite infection, activation of B cell receptors that produced second messengers, ligand binding and uptake by scavenger receptors, regulation of BCRs through CD22, and interactions between cells on the vascular wall (Figure S5A,B). Furthermore, analysis revealed that pathways enriched in the high‐risk individuals dealt primarily with cilium organization, epidermis development, microtubule bundle formation, microtubule cytoskeleton organization, and cilium when we analyzed GO gene sets. Supporting Figure 5C,D, pathways significantly enriched in the low‐risk group were primarily involved in immune activation, adaptive immunological response, somatic recombination of immune receptors, and both the activation and differentiation of alpha beta T cells. These results provided more evidence that pathways involving the immune response may contribute to the good prognoses seen in HCC patients with low immLysoS scores.

### 3.8. Correlation of ImmLysoS With the TIME

We utilized ssGSEA to determine the correlation between immLysoS scores and immune cell infiltration. This allowed us to evaluate whether the prognostic guidance given by immLysoS is linked to the TIME. Immune cell infiltration levels were negatively correlated with immLysoS scores (Figure S6A,B). Immune cell types known to promote tumor killing showed a particularly strong negative connection with immLysoS (Figure S6C–E). Beyond that, further analysis using ssGSEA revealed a significant inverse correlation between immLysoS scores and the expression of 46 immune checkpoint genes. Low immLysoS scores were positively associated with HCC patients’ prognosis, which may be explained by an immunological milieu where tumor‐killing immune cells are highly expressed and immune checkpoint genes are not expressed as much.

### 3.9. The Predictive Value of ImmLysoS in Immunotherapy and Targeted Therapy

To explore the potential of immLysoS in guiding clinical decisions regarding immunotherapy and drug therapy, we first compared the immunophenotype scores (IPSs) for two ICIs (PD‐1 and CTLA‐4) across two immunotherapy groups based on high and low immLysoS scores (Figure S7A–D). We also used information from the IMvigor210 cohort, which included 348 samples treated with PD‐L1, to confirm that immunotherapy was effective. Patients in this cohort with lower immLysoS scores had improved survival rates, suggesting that these scores significantly predicted prognosis (Figure S7E). The results thus revealed a promising role for immLysoS in predicting responses to immunotherapy among HCC patients. Finally, we performed a drug sensitivity analysis of sorafenib, a first‐line treatment for HCC, based on immLysoS, and these results indicated that different subgroups of patients based on immLysoS score exhibited varying susceptibilities to sorafenib treatment, with the low immLysoS score group showing better treatment outcomes. In addition, CCK‐8 cell experiments targeting sorafenib further validated our preliminary bioinformatics predictions (Figure S7F–H).

### 3.10. Knockdown of RGS2

RGS2, one of the genes utilized to construct the immLysoS, was uniquely associated with poorer prognosis in HCC when highly expressed (Figure 5A). The existing literature suggests a potential role for RGS2 in the malignant progression of certain cancers, although its specific function in HCC remains unexplored [26–28]. Therefore, we investigated the biological role of RGS2 in HCC.

We began by examining the differential expression of the four model genes in normal human hepatocytes (LX2) and HCC cells (Hep3B and MHCC97H). Significantly, only RGS2 exhibited substantial upregulation in both Hep3B and MHCC97H cells in comparison to LX2 cells (Figure 8A,B). Subsequently, Hep3B and MHCC97H cells were effectively transfected with RGS2‐specific shRNA (sh‐RGS2), which resulted in efficient knockdown (Figure 8C,D). We then employed CCK‐8, colony formation, transwell, and wound healing assays to determine the impact of RGS2 knockdown on the proliferation and invasion of HCC cells.

Figure 8Knockdown of RGS2 inhibits the proliferation and migration of HCC cell lines. (A) The mRNA expression levels of the four key immLysoS genes (GZMH, KLRB1, RGS2, and SLC6A1) in normal human hepatocytes (LX2) and HCC cells (Hep3B and MHCC97H) were determined by RT‐qPCR analysis. (B) The protein expression levels of the four key immLysoS genes in LX2, Hep3B, and MHCC97H cells were detected by Western blotting. (C, D) The knockdown efficiency of RGS2 in Hep3B and MHCC97H cells transfected with two independent RGS2‐specific shRNAs (sh‐RGS2#1 and sh‐RGS2#2) was confirmed by Western blotting, compared to a negative control shRNA (sh‐NC). (E, F) Cell Counting Kit‐8 (CCK‐8) assays demonstrated that the proliferative capacity of HCC cells was significantly attenuated upon RGS2 knockdown compared to the sh‐NC group. (G) Colony formation assays revealed that the clonogenic survival of HCC cells was markedly suppressed following RGS2 depletion. (H, I) Transwell migration assays showed a significant reduction in the migratory ability of HCC cells after RGS2 knockdown compared to control cells. (J, K) Wound healing assays indicated that RGS2 silencing impeded the migration and wound closure capacity of HCC cells. (L–N) Tumor volume and weight of xenograft tumors formed by the indicated Hep3B cells. Data are presented as mean ± SD.  ^∗^ *p* < 0.05;  ^∗∗^ *p* < 0.01;  ^∗∗∗^ *p* < 0.001;  ^∗∗∗∗^ *p* < 0.0001. (All biological experiments were performed in triplicate. The scratch wound length was quantified using ImageJ software, and statistical differences between groups were assessed using *t*‐tests.)

**Figure:**
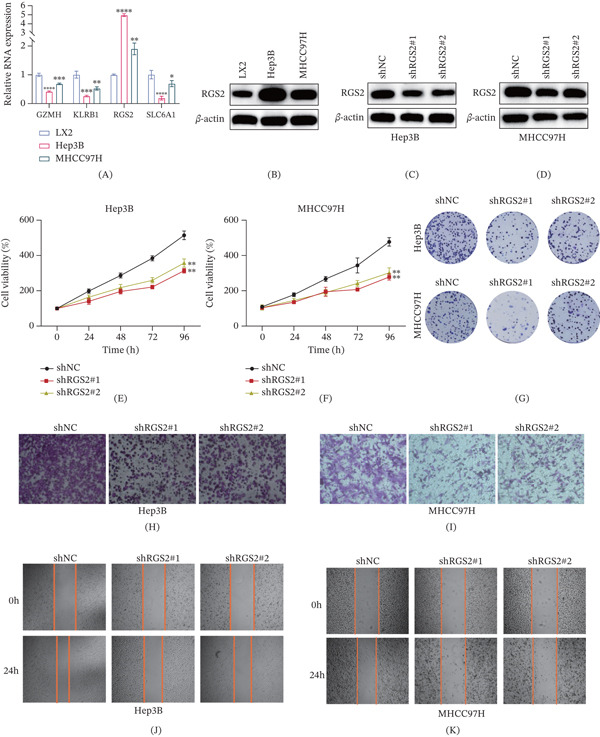
(A)

**Figure:**
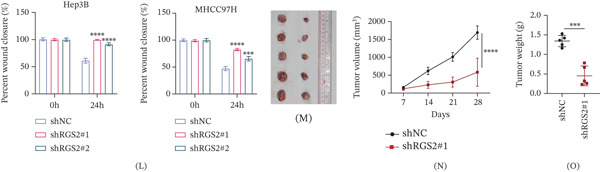
(B)

The experiments indicated that RGS2 knockdown markedly suppressed the proliferation of HCC cells (Figure 8E–G and Figure S8A). Moreover, in vitro, knockdown of RGS2 significantly inhibited the migratory and wound healing activities of HCC cells (Figure 8H–K and Figure S8B). In vivo, knockdown of RGS2 significantly inhibited HCC tumor volume and weight (Figure 8L–N). Immunohistochemical (IHC) staining for RGS2 and Ki‐67 was conducted on tumor tissues derived from the two groups of mice. The results demonstrated that knockdown of RGS2 significantly reduced the expression levels of both RGS2 and Ki‐67 (Figure S8C,D). These results suggest that RGS2 facilitates the proliferation and metastasis of HCC.

## 4. Discussion

The incidence of liver cancer has shown an increasing trend [29], and the great majority of diagnoses and fatalities from liver cancer (45%) are due to HCC. Patients at the early stage now survive longer than ever before, thanks to new diagnostic and treatment methods, although OS for liver cancer is still low [30]. However, immunotherapy, for example, does not work for everyone, and this may have something to do with variations in TIME across HCC patients. Therefore, the active search for novel and validated prognostic risk scoring models in HCC can be of great benefit in improving patient prognosis and tailoring personalized treatment regimens.

Furthermore, recent studies have elucidated a partial link between lysosomes and disease [31, 32]. This brought to our attention a specific type of lysosome: SL. SL is a class of organelles that possesses both the traditional lysosomal function of degrading and recycling biomolecules and the function of acting as a storage and transport vehicle for effector molecules [33]. Immune cells like NK cells can eliminate tumor cells and cells infected with viruses by releasing cytolytic granules through SLs [34]. We postulate, based on prior research, that SL controls HCC growth and tumor immunity in the TIME of HCC. Since no prior research has systematically examined the prognostic significance and assessment of immunotherapeutic outcomes in HCC, we felt compelled to fill that void.

The first step of our research was to extract 13 genes associated with SL from the MsigDB database and name them immLysorg. We made sure that these 13 immLysorgs were expressed differently in HCC cancer and paracancer. Then, we looked at the mutation status and frequency of CNVs of immLysorgs in HCC. Univariate Cox regression showed that 12 among them were substantially related to OS. In particular, the C2 subgroup was associated with a more favorable prognosis, more immune cell infiltration, and more pathways related to immune boosting. We then identified 417 DEGs between the two SL subtypes. DEGs were found to be more abundant in several immune pathways according to our enrichment analysis. They included pathways that involve intracellular immune responses and immune signaling, antigen binding and immunoglobulin receptor binding, chemokine pathways, cytotoxicity mediated by NK cells, and antigen processing and presentation. Afterwards, we analyzed DEGs in the C1 and C2 subgroups using RF and NN algorithms and selected five genes, NKG7, GZMH, CST7, PRF1, and GZMB, as being particularly important for differentiating C1 and C2 subgroups. This suggested that the prognostic differences among HCC patient subgroups may primarily stem from the differential expression of these five genes.

Further, 39 prognostically relevant DEPGs were identified, and two genotypes were additionally established according to their expression profiles: A and B. The prognosis was worse for Genotype B, and there was a marked difference in the immLysorgs expression profiles between the two genotypes. To evaluate the status of SL in HCC patients, we screened 32 protein‐coding genes from DEPGs and selected four important genes (GZMH, KLRB1, RGS2, and SLC6A1) using LASSO and multivariate Cox regression. ImmLysoS was developed utilizing these four genes, and possible molecular subtypes were found to predict HCC prognosis better. Next, we fully assessed the utility of immLysoS in HCC in predicting prognosis and further analyzed its prognostic relevance among different clinical subgroups. We measured its sensitivity and specificity in predicting OS and PFS in both TCGA and GEO validation sets, as well as its ability to predict OS in HCC groups at early and late stages and low and high grades.

In the extant literature, it is reported that copy number deletion of the GZMH gene is related to poor prognosis in nasopharyngeal carcinoma [35]. Similarly, KLRB1 expression is closely associated with glioma molecular pathology, and its unique role in T cell dysfunction promotes glioma progression and evolution [36]. Likewise, RGS2 enables non–small cell lung cancer to exhibit a predominantly dormant‐like phenotype and high viability in a hostile microenvironment [28, 37]. Finally, overexpression of SLC6A1 in prostate cancer has been linked to resistance to doxorubicin treatment, rapid tumor growth, and a low biochemical recurrence‐free survival rate [38]. The results of these investigations all lend credence to our novel immLysoS concept.

Looking at the variations in ESTIMATE score, RNAs, gene mutation frequency, and TMB between groups with high and low immLysoS from a TME perspective allowed us to begin exploring the potential reasons why immLysoS can differentiate the prognosis of HCC. In contrast to the low immLysoS group with high ESTIMATE scores and low RNA levels, we discovered that the immune stromal component of the TME and RNAs in the high immLysoS group was distinct. With lower scores indicating less tumor purity and worse prognosis, the ESTIMATE score can imply the ratio of stromal to immune cells in the TME [39]. A worse prognosis is suggested by a greater RNA score and a larger amount of tumor dedifferentiation, which are tightly connected to one another [25]. Notably, there were disparities in gene mutation rates between the two groups stratified by immLysoS scores, including TP53 [40], CTNNB1 [40], TTN [41], LRP1B [42], and OBSCN [43].

To understand the possible biological functions and pathways underlying the prognostic differences associated with immLysoS, we conducted GSEA between the immLysoS‐based subgroups. The results implicated immune response–related pathways in the favorable prognosis of the low‐score group. This was consistent with the previous statement that SL is primarily linked to immune response and signaling pathways of the tumor [16, 17].

Hence, we proceeded to investigate the connection between immLysoS and TIME. We observed that immLysoS scores exhibited a highly significant negative correlation with the populations of immune cells that support tumor killing, such as activated CD8 T cells and activated B cells. When activated CD8 T cells and B cells infiltrate the TME, they can interact functionally to boost local immunological effects, which in turn destroy tumor cells and improve patient prognosis [44, 45]. A key indicator of the development of HCC is a decrease in NK cells [46]. Soon, advanced HCC patients will be able to participate in a Phase II clinical trial (NCT02008929) that will assess the safety and effectiveness of an ex vivo expanded allogeneic NK cell (MG4101) as a subsequent treatment after curative liver resection. Such patients have shown improvement in response rates to ICIs in the last 10 years [47, 48].

The restrictions imposed by the tumor immune state likely account for the low response rate of immunotherapy in HCC patients [49]. To find out if immLysoS could predict how well anticancer immunotherapy would work, we looked at the expression of 46 common immune checkpoints in both groups. Most of these tests were found to be negatively associated with immLysoS scores.

To reveal the practical implications of immLysoS to guide clinical treatment, we used the immunotherapy cohort from TCIA data and the IMvigor210 PD‐L1 treatment cohort for bladder cancer to validate the immunotherapy efficacy in groups with high and low immLysoS scores. We found that immLysoS can be a good predictor of whether patients with liver cancer benefit from immunotherapy. The current systemic therapy for advanced HCC is mainly based on target‐free combinations, and the clinical significance of sorafenib as the most classical targeted drug for HCC treatment is very important [50]. Thus, we also investigated whether there was a connection between immLysoS and the sensitivity of sorafenib treatment and discovered a negative correlation between them. Based on what we know so far, the immLysoS that was built using SL in this study can help with clinical treatment stratification for HCC patients. The possible mechanisms could be related to factors such as tumor purity, RNAs, the frequency of oncogene TP53 and CTNNB1 mutations, and sensitivity to the targeted drug sorafenib. Both our in vitro and in vivo experiments proved that RGS2 promotes HCC growth and metastasis.

There are a number of caveats to our study that we need to address. One caveat is that there may be case selection bias because we used publicly available data in our analysis. In addition, numerous factors influence immune infiltration, including tumor heterogeneity, TMB, microsatellite instability, tumor histology type, intrinsic factors of immune cells, and the diverse methodologies used for immune cell infiltration analysis. Although we utilized a substantial number of external datasets to corroborate our findings, further validation with large‐scale clinical case data is still required to confirm the authenticity of our results. Although immLysoS may have significant predictive value in HCC, its underlying mechanisms remain unexplored and warrant further investigation through additional clinical studies. Therefore, a primary goal of future research will be to figure out the molecular mechanisms responsible for the tumor‐promoting functions of RGS2 in HCC.

## 5. Conclusions

We constructed a gene signature based on 13 known immLysorgs for the purpose of prognostic risk stratification and prediction of responses to immunotherapy and targeted therapy in HCC, both of which were found to be highly accurate. Additionally, we investigated the immunological significance, association with drug sensitivity, and biological functions of immLysoS in HCC pathogenesis, due to its potential importance for personalized prognostic monitoring.

## Author Contributions

D.L., Zhit.Y., X.L., and Jia.L. designed and initiated this study. Zhip.Y. and B.T. performed the experiments. Zhip.Y., H.C., and Y.Z. performed the bioinformatics analysis. Zhip.Y., Jie.L., and Jia.L. wrote the manuscript. Zhip.Y., B.T., and Y.Z. have contributed to the work equally and should be regarded as co‐first authors.

## Funding

This research was supported by grants from Guangzhou Women and Childrens Medical Center Clinical Doctor Initiation Scientific Research Fund (Grant No. 2024BS003 to Zhip.Y. and Grant No. 2024BS016 to D.L.) and Guangzhou Science and Technology Plan Projects (Grant Nos. 2025A03J4374 and 2025A04J4616 to Zhit.Y.).

## Disclosure

All authors read and approved the final manuscript.

## Ethics Statement

All analyses based on publicly available datasets did not require separate ethical approval. Animal experiments were conducted in accordance with the guidelines approved by the Institutional Animal Care and Use Committee of the Qite Biotech Animal Experiment Center, Guangzhou, China (Protocol No. QT 20260112001). All efforts were made to minimize animal suffering and to use the minimum number of animals necessary to achieve reliable scientific outcomes.

## Consent

The authors have nothing to report.

## Conflicts of Interest

The authors declare no conflicts of interest.

## Supporting information


**Supporting Information 1** Additional supporting information can be found online in the Supporting Information section. Supporting Information. Figure S1: Expression and genetic alteration of immLysorgs in HCC. (A) The expression of 13 immLysorgs in HCC and normal tissues. (B–D) The mutation frequency and CNV and chromosomal localization of 13 immLysorgs in HCC.  ^∗^ *p* < 0.05,  ^∗∗^ *p* < 0.01, and  ^∗∗∗^ *p* < 0.001; ns, not statistically different; immLysorgs, immune lysosome‐related genes. Supporting Information 2 Figure S2. Prognostic significance of immLysorgs of HCC patients in TCGA. (A–L) Single‐gene K‐M survival analysis based on the TCGA‐LIGC cohort showing that the expression of 12 immLysorgs significantly affects patient prognosis. (M) Correlation prognostic network based on the TCGA‐LIGC cohort consisting of 13 immLysorgs. Each sphere represents the Cox test for a given gene, and the linkage between spheres represents the correlation between genes. immLysorgs, immune lysosome‐related genes. Supporting Information 3 Figure S3: GSVA heat map showing the differences in pathways in the two clusters. (A) Gene set from “c2.cp.kegg.v7.5.1.symbols.gmt.” (B) Gene set from “c2.cp.reactome.v7.5.1.symbols.gmt.” (C) Gene set from “h.all.v7.5.1.symbols.gmt.” Supporting Information 4 Figure S4: Functional enrichment analysis of DEGs between C1 and C2 subgroups. (A) Volcano map of DEGs. (B, D) Analysis of GO‐enriched BP, CC, and MF terms demonstrating the possible role of DEGs. (C, E) Kyoto Encyclopedia of Genes and Genomes (KEGG) pathway enrichment analysis revealing possible pathways. Supporting Information 5 Figure S5: GSEA analysis of differential genes between different immLysoS groups. (A, B) GSEA analysis between high‐ and low‐risk groups using the gene set “c2.cp.reactome.v7.5.1.symbols.gmt.” (C, D) GSEA analysis between high‐ and low‐risk groups using gene set “c5.go.v7.4.symbols.gmt.” Supporting Information 6 Figure S6: Correlation of immLysoS with the tumor immune microenvironment. (A, B) Correlation of immLysoS score with the level of infiltration of 23 immune cells. (C–E) Relationship of immLysoS score with the population of immune cells promoting tumor killing action: (C) activated CD8 T cells, (D) activated B cells, and (E) NK cells. (F) Correlation of immLysoS score with the level of 46 immune checkpoint genes epi‐oh. ImmLysoS, immune lysosome score;  ^∗^ *p* < 0.05;  ^∗∗^ *p* < 0.01;  ^∗∗∗^ *p* < 0.001. Supporting Information 7 Figure S7: The role of immLysoS in predicting the efficacy of immunotherapy and targeted drug therapy. (A–D) Comparison of four immune phenotype score IPSs between different immLysoS groups. (E) KM curves between different immLysoS groups in the IMvigor210 cohort. (F, G) The relationship between immLysoS and susceptibility to treatment with sorafenib. ImmLysoS, immune lysosome score. (H) Sorafenib sensitivity assay in Hep3B and MHCC97H cells ( ^∗^ *p* < 0.05;  ^∗∗^ *p* < 0.01). Supporting Information 8 Figure S8: Statistical analysis and immunohistochemical staining of mouse tumors. (A) Statistical analysis of colony formation assays in Hep3B and MHCC97H cells. (B) Statistical analysis of transwell migration assays in Hep3B and MHCC97H cells. (C). Immunohistochemical staining of RGS2 in mouse tumor tissues. (D) Immunohistochemical staining of Ki‐67 in mouse tumor tissues. Scale bars: 50 *μ*m (20× magnification) and 20 *μ*m (40× magnification).

## Data Availability

All the original data and codes in this article have been uploaded to NutCloud and can be accessed through the following link: https://www.jianguoyun.com/p/DVcfDkoQjNLPChiUpp0GIAA. Full uncropped gel and blot images are available in Supporting Information.
