# Supplementary material for: Secretory Lysosome‐Related Gene Signature Defines the Immune Microenvironment and Identifies RGS2 as a Prometastatic Factor in Hepatocellular Carcinoma
Source: Hum Mutat. 2026 May 16;2026:3501996. doi: 10.1155/humu/3501996 (PMC13179717; doi:10.1155/humu/3501996)
Supplement: Supplementary file 1 — Supporting Information 1 Additional supporting information can be found online in the Supporting Information section. Supporting Information. Figure S1: Expression and genetic alteration of immLysorgs in HCC. (A) The expression of 13 immLysorgs in HCC and normal tissues. (B–D) The mutation frequency and CNV and chromosomal localization of 13 immLysorgs in HCC. ∗ p < 0.05, ∗∗ p < 0.01, and ∗∗∗ p < 0.001; ns, not statistically different; immLysorgs, immune lysosome‐related genes. Supporting Information 2 Figure S2. Prognostic significance of immLysorgs of HCC patients in TCGA. (A–L) Single‐gene K‐M survival analysis based on the TCGA‐LIGC cohort showing that the expression of 12 immLysorgs significantly affects patient prognosis. (M) Correlation prognostic network based on the TCGA‐LIGC cohort consisting of 13 immLysorgs. Each sphere represents the Cox test for a given gene, and the linkage between spheres represents the correlation between genes. immLysorgs, immune lysosome‐related genes. Supporting Information 3 Figure S3: GSVA heat map showing the differences in pathways in the two clusters. (A) Gene set from “c2.cp.kegg.v7.5.1.symbols.gmt.” (B) Gene set from “c2.cp.reactome.v7.5.1.symbols.gmt.” (C) Gene set from “h.all.v7.5.1.symbols.gmt.” Supporting Information 4 Figure S4: Functional enrichment analysis of DEGs between C1 and C2 subgroups. (A) Volcano map of DEGs. (B, D) Analysis of GO‐enriched BP, CC, and MF terms demonstrating the possible role of DEGs. (C, E) Kyoto Encyclopedia of Genes and Genomes (KEGG) pathway enrichment analysis revealing possible pathways. Supporting Information 5 Figure S5: GSEA analysis of differential genes between different immLysoS groups. (A, B) GSEA analysis between high‐ and low‐risk groups using the gene set “c2.cp.reactome.v7.5.1.symbols.gmt.” (C, D) GSEA analysis between high‐ and low‐risk groups using gene set “c5.go.v7.4.symbols.gmt.” Supporting Information 6 Figure S6: Correlation of immLysoS with the tumor immune microenvi [file HUMU-2026-3501996-s001.zip › Supplementary_Material_revised - V1.docx]

Secretory Lysosome-Related Gene Signature Defines the Immune Microenvironment and Identifies RGS2 as a Pro-Metastatic Factor in Hepatocellular Carcinoma

**Zhipeng Ye^1^**†**, BuLang Tang^2†*^, Yuanjian Zhang^3†^, Hanhan Chen^3,4^, Jie Li^3^, Zhitao Ye^3^, Xuejian Liu^5#^, Jiaxi Li^6#^, Di Li^7#^**

^1^Hepatology Unit, Departments of Infectious Disease, Guangzhou Women and Children's Medical Center, Guangzhou Medical University, Guangzhou, 510623, China.

^2^The Fifth Affiliated Hospital of Guangzhou Medical University, Guangzhou, 510623, China.

^3^Guangzhou Women and Children's Medical Center, Guangzhou Medical University, Guangzhou, 510623, China.

^4^School of Pharmaceutical Sciences, Guangzhou Medical University, Guangzhou, 511436, China.

^5^Department of Radiology, Xinjiang 474 Hospital, Urumqi, China

^6^Department of Clinical Laboratory, Xintang Hospital, Guangzhou, 511436, China.

^7^Department of Ultrasonography, Guangzhou Women and Children's Medical Center, Guangzhou Medical University, Guangzhou, 510623, China.

**Zhipeng Ye^1^**†**, BuLang Tang^2†*^, Yuanjian Zhang^3†^**

† These authors have contributed equally to this work

The manuscript contains 2863 words in total.

*** Correspondence:**Xuejian Liu, Department of Radiology, Xinjiang 474 Hospital, Urumqi, China. Email: xuejian1991@foxmail.com,

Jiaxi Li, Department of Clinical Laboratory, Xintang Hospital, Guangzhou, 510623, China. Email: 547562270@qq.com.

Di Li, Department of Ultrasonography, Guangzhou Women and Children's Medical Center, Guangzhou Medical University, Guangzhou, 510623, China. Email: lidi8@alumni.sysu.edu.cn.
[email@uni.edu](mailto:email@uni.edu)

Supplementary Material


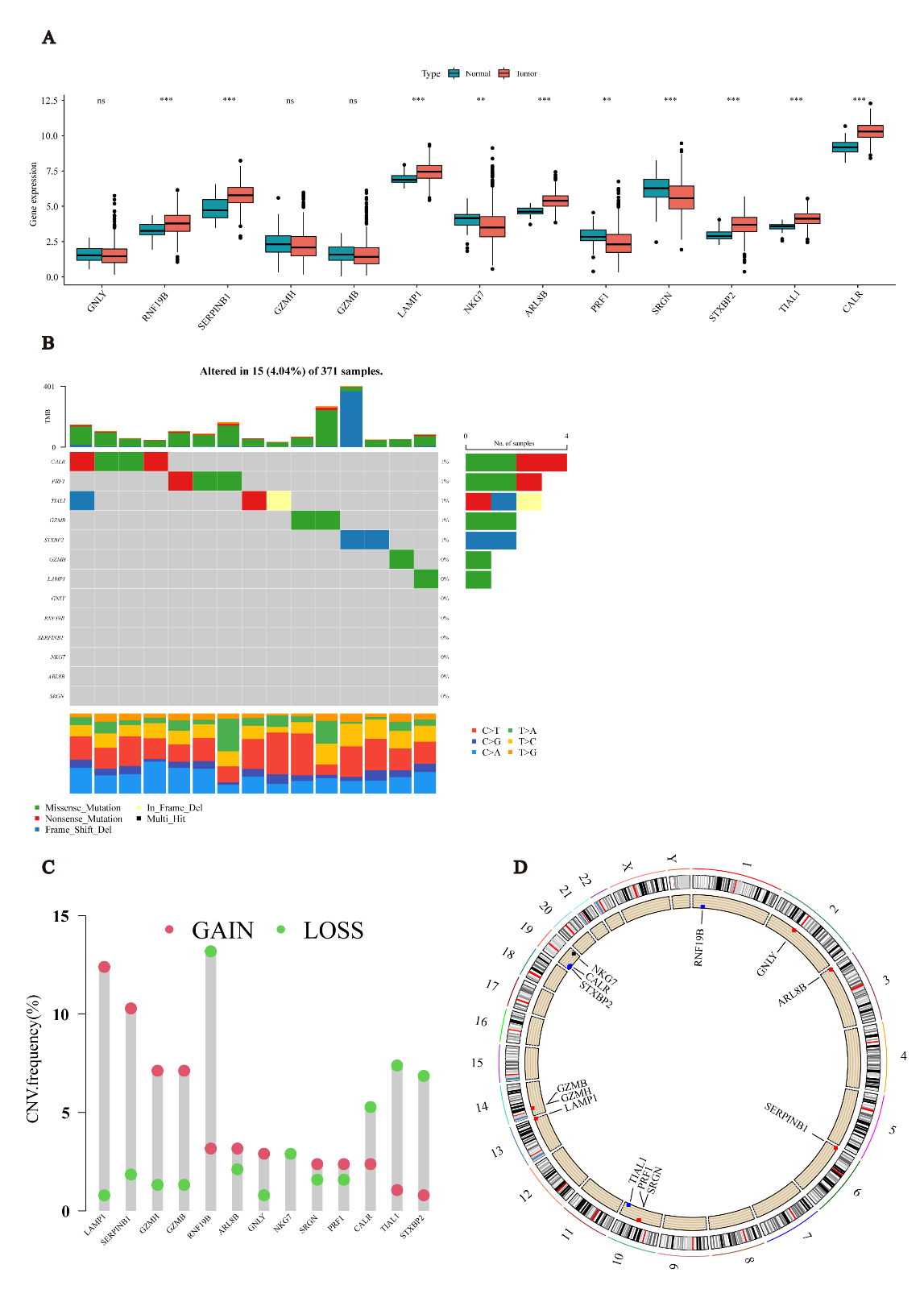


**Supplementary Figure 1.** Expression and genetic alteration of immLysorgs in HCC. (A) the expression of 13 immLysorgs in HCC and normal tissues. (B-D) The mutation frequency and CNV and chromosomal localization of 13 immLysorgs in HCC. **p* < 0.05, ** *p* <0.01, *** *p* <0.001; ns: not statistically different. immLysorgs, immune lysosome-related genes.


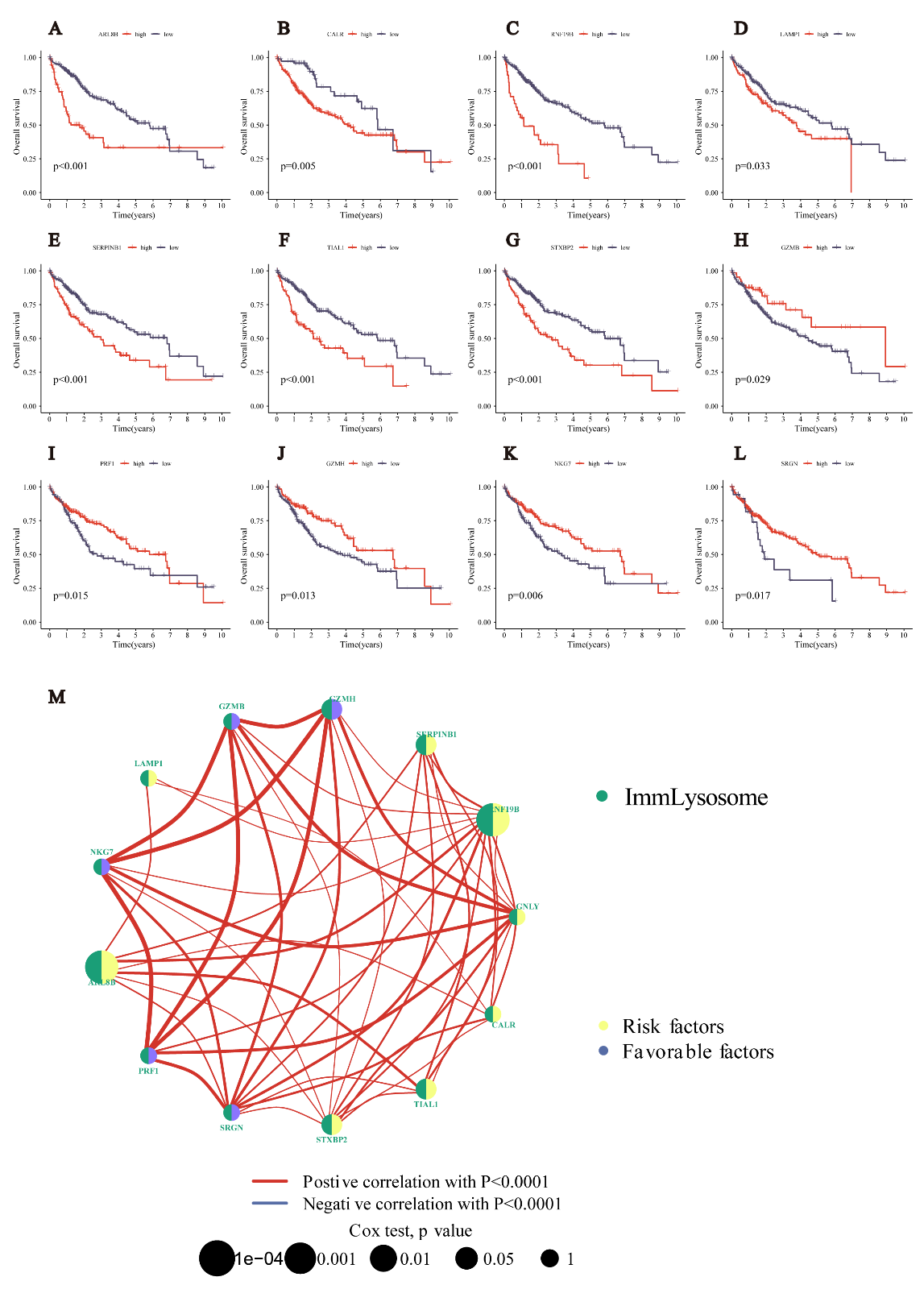


**Supplementary Figure 2.** Prognosis significance of immLysorgs of HCC patients in TCGA. (A-L) Single-gene K-M survival analysis based on TCGA-LIGC cohort showing that the expression of 12 immLysorgs significantly affects patient prognosis. (M) Correlation prognostic network based on TCGA-LIGC cohort consisting of 13 immLysorgs. Each sphere represents the Cox test for a given gene, and the linkage between spheres represents the correlation between genes. ImmLysorgs: immune lysosome-related genes.


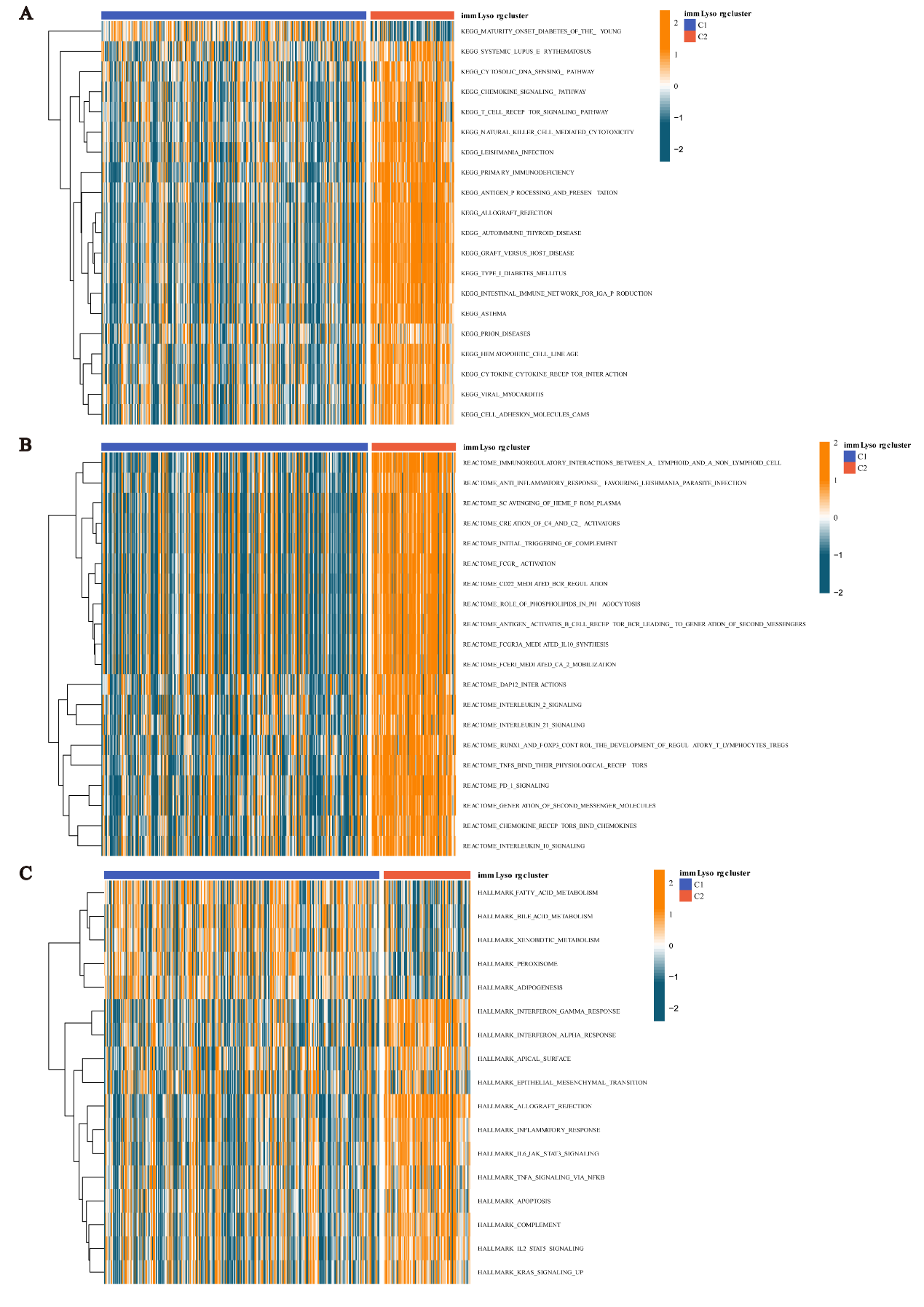


**Supplementary Figure 3.** GSVA heat map showing the differences in pathways in the two clusters. (A) Gene set from "c2.cp.kegg.v7.5.1.symbols.gmt". (B) Gene set from "c2.cp.reactome.v7.5.1.symbols.gmt". (C) Gene set from "h.all.v7.5.1.symbols.gmt".


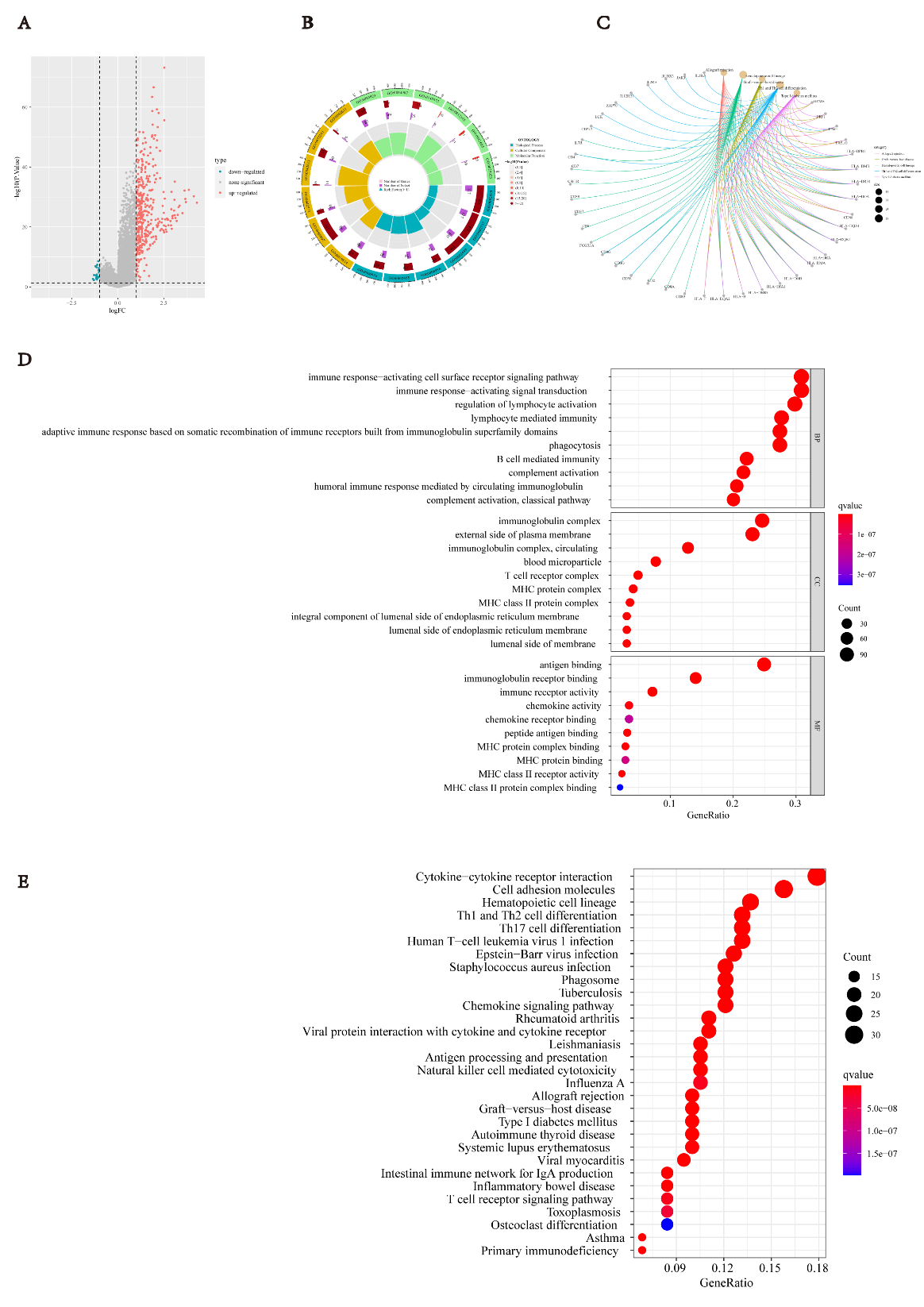


**Supplementary Figure 4.** Functional enrichment analysis of DEGs between C1 and C2 subgroups. (A) Volcano map of DEGs. (B, D) Analysis of GO-enriched BP, CC, and MF terms demonstrating the possible role of DEGs. (C, E) Kyoto Encyclopedia of Genes and Genomes (KEGG) pathway enrichment analysis revealing possible pathways.


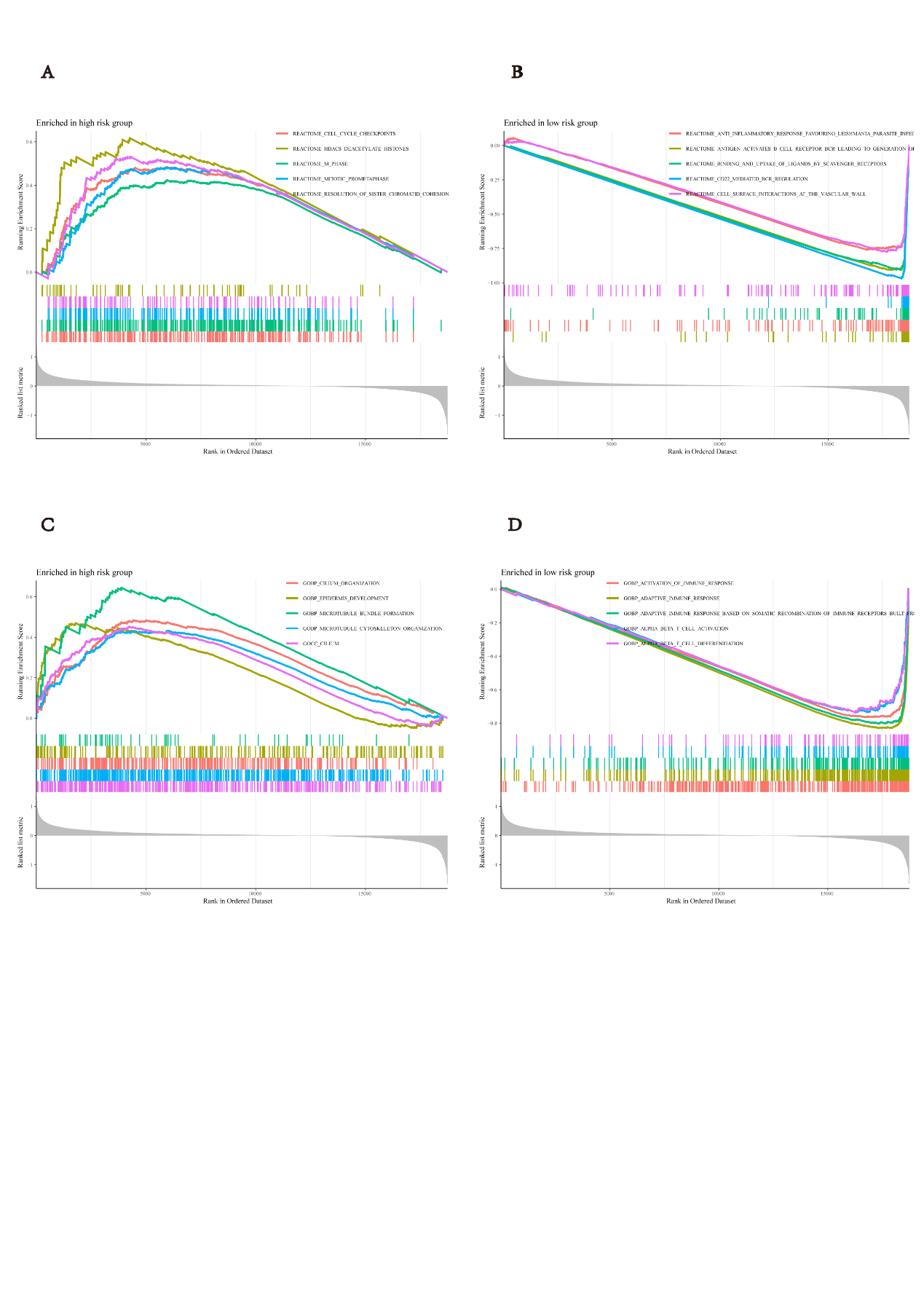


**Supplementary Figure 5.** GSEA analysis of differential genes between different immLysoS groups. (A-B) GSEA analysis between high and low risk groups using the gene set "c2.cp.reactome.v7.5.1.symbols.gmt". (C-D) GSEA analysis between high and low risk groups using gene set "c5.go.v7.4.symbols.gmt".


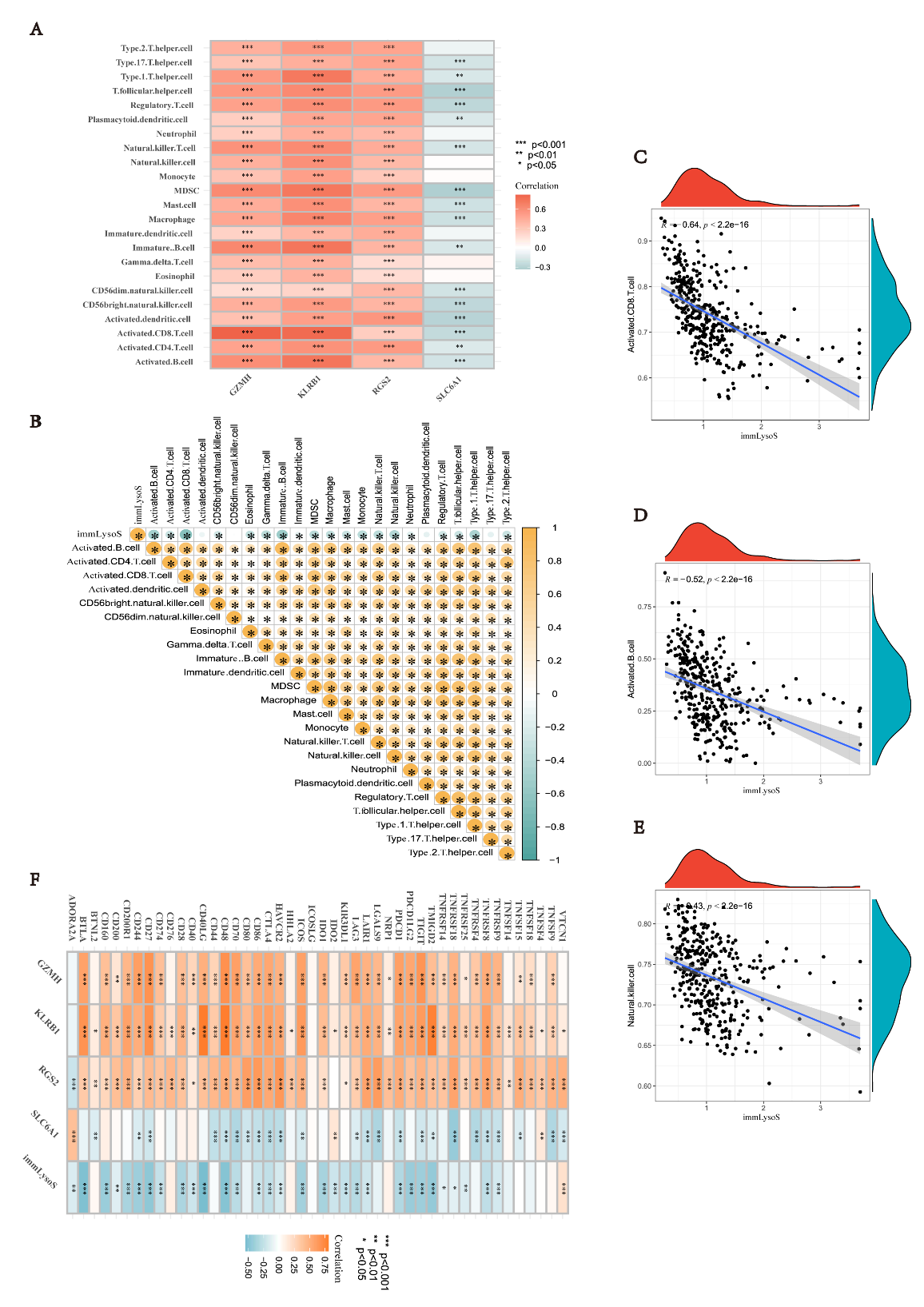


**Supplementary Figure 6.** Correlation of immLysoS with the tumor immune microenvironment. (A-B) Correlation of immLysoS score with the level of infiltration of 23 immune cells. (C-E) Relationship of immLysoS score with the population of immune cells promoting tumor killing action; (C) activated CD8 T cells, (D) activated B cells, (E) NK cells. (F) Correlation of immLysoS score with the level of 46 immune checkpoint genes epi-oh. ImmLysoS: immune Lysosome score; **p* < 0.05; ***p* < 0.01; and ****p* < 0.001.


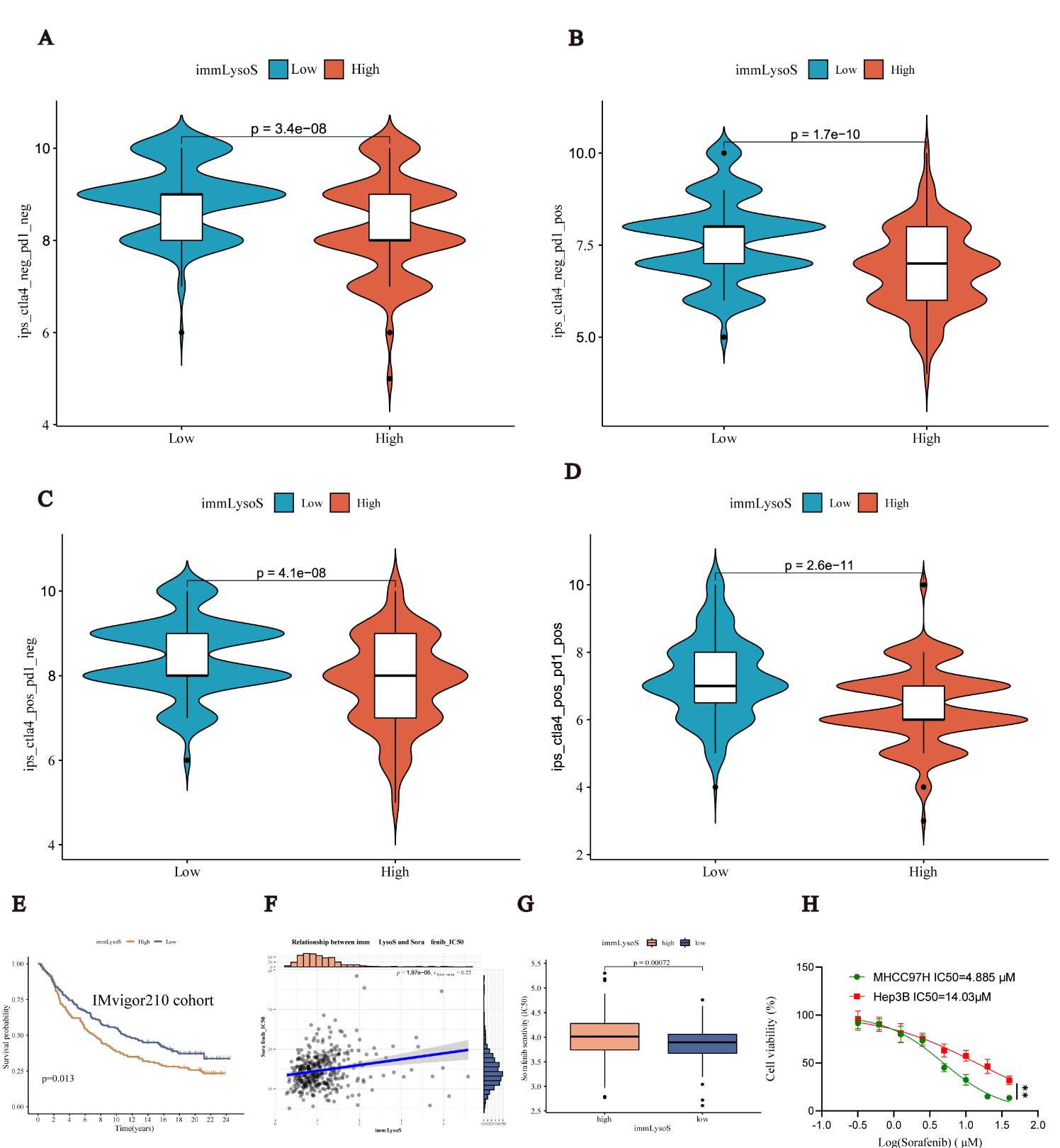


**Supplementary Figure 7.** The role of immLysoS in predicting the efficacy of immunotherapy and targeted drug therapy. (A-D) Comparison of 4 immune phenotype score IPSs between different immLysoS groups. (E) KM curves between different immLysoS groups in the IMvigor210 cohort. (F-G) The relationship between immLysoS and susceptibility to treatment with sorafenib. ImmLysoS: immune lysosome score. (H) Sorafenib sensitivity assay in Hep3B and MHCC97H cells. (*P<0.05, **P<0.01)


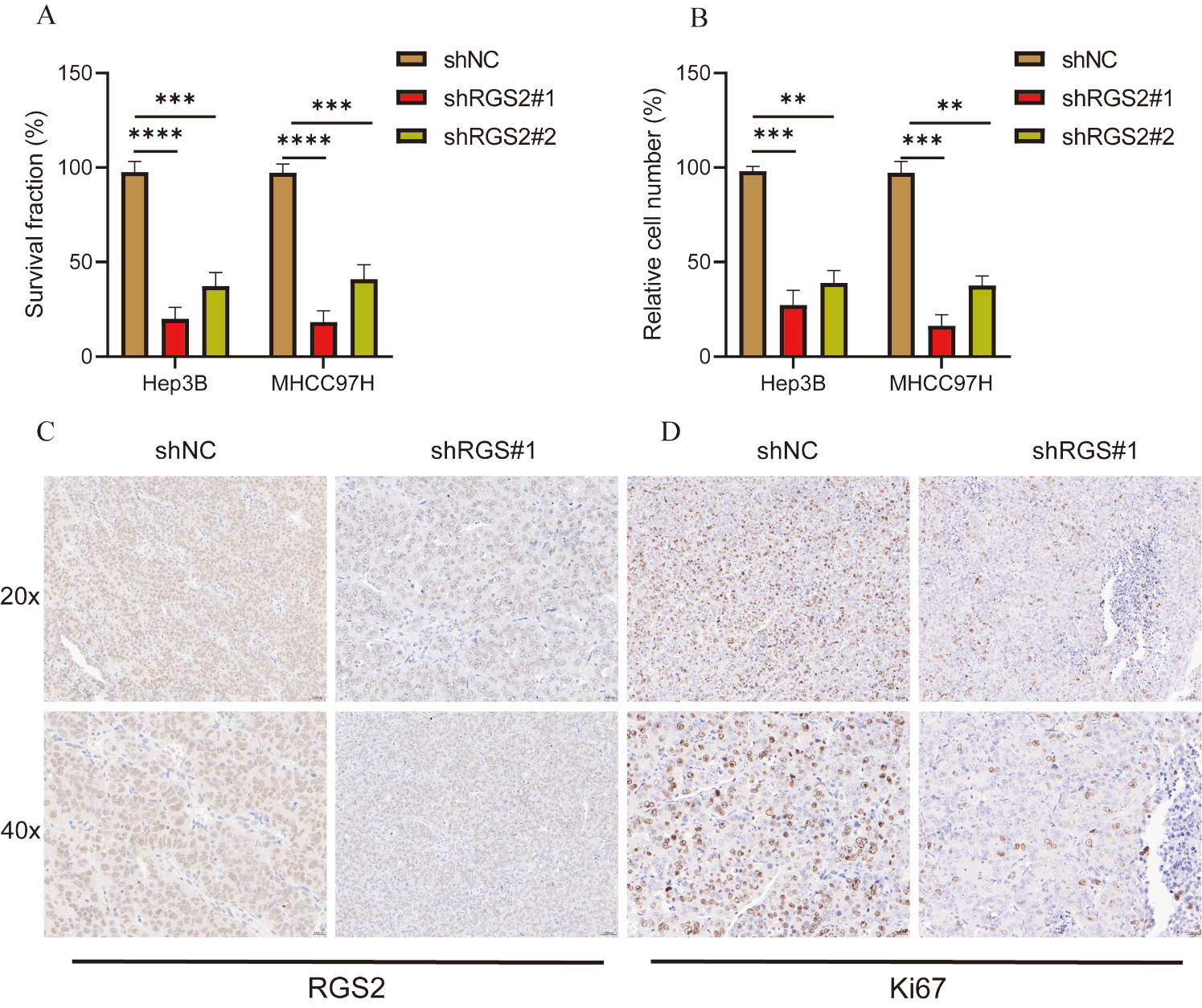


**Supplementary Figure 8.** Statistical analysis and immunohistochemical staining of mouse tumors. (A) Statistical analysis of colony formation assays in Hep3B and MHCC97H cells. (B) Statistical analysis of Transwell migration assays in Hep3B and MHCC97H cells. (C). Immunohistochemical staining of RGS2 in mouse tumor tissues. (D) Immunohistochemical staining of Ki-67 in mouse tumor tissues. Scale bars: 50 μm (20× magnification), 20 μm (40× magnification).
