# Supplementary figures and images for: Secretory Lysosome‐Related Gene Signature Defines the Immune Microenvironment and Identifies RGS2 as a Prometastatic Factor in Hepatocellular Carcinoma
Source: Hum Mutat. 2026 May 16;2026:3501996. doi: 10.1155/humu/3501996 (PMC13179717; doi:10.1155/humu/3501996)

**A**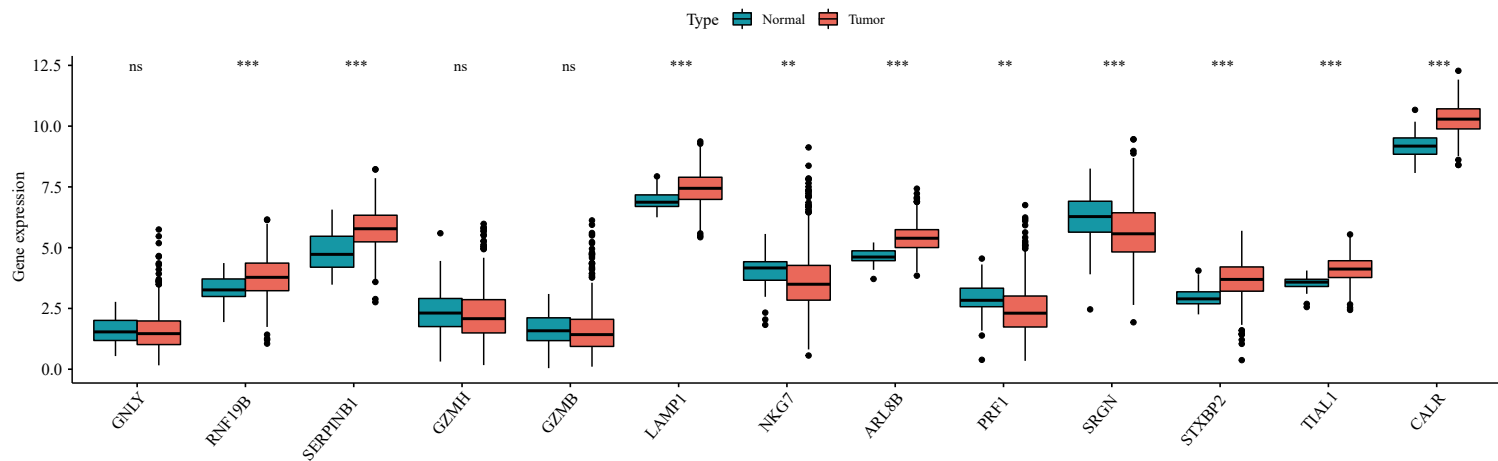**B**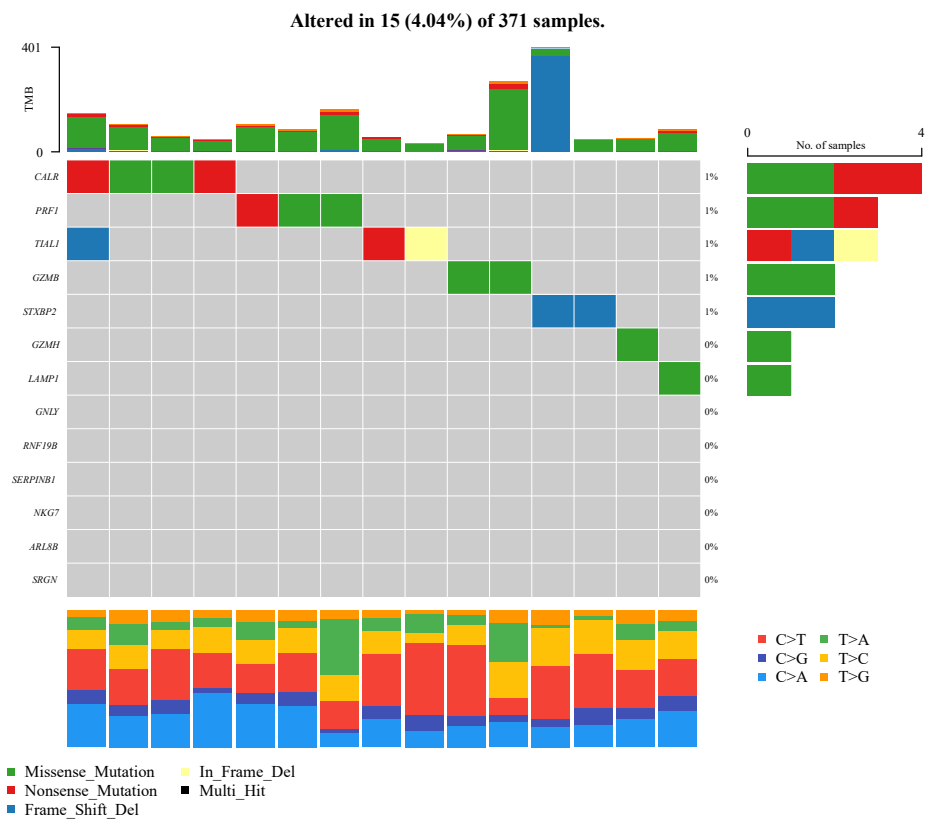**C**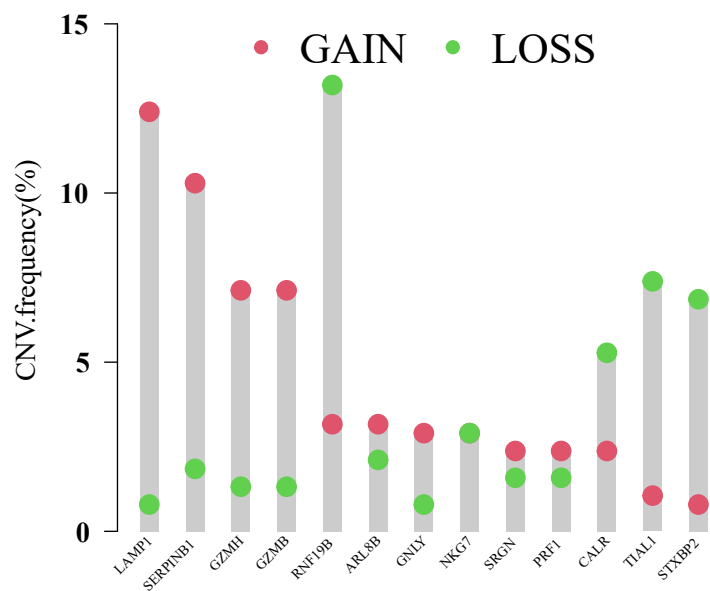**D**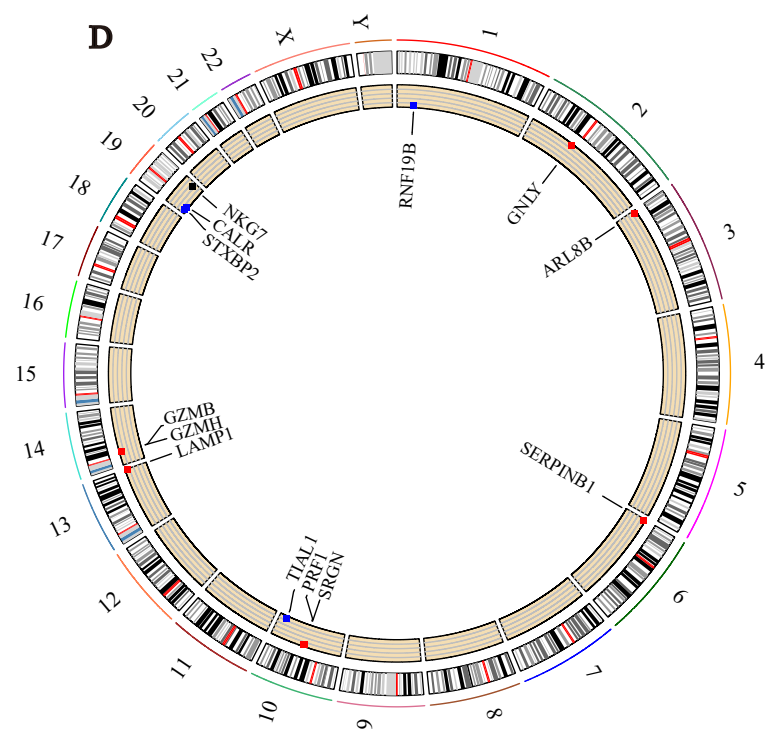

Supplement: Supplementary file 1 — Supporting Information 1 Additional supporting information can be found online in the Supporting Information section. Supporting Information. Figure S1: Expression and genetic alteration of immLysorgs in HCC. (A) The expression of 13 immLysorgs in HCC and normal tissues. (B–D) The mutation frequency and CNV and chromosomal localization of 13 immLysorgs in HCC. ∗ p < 0.05, ∗∗ p < 0.01, and ∗∗∗ p < 0.001; ns, not statistically different; immLysorgs, immune lysosome‐related genes. Supporting Information 2 Figure S2. Prognostic significance of immLysorgs of HCC patients in TCGA. (A–L) Single‐gene K‐M survival analysis based on the TCGA‐LIGC cohort showing that the expression of 12 immLysorgs significantly affects patient prognosis. (M) Correlation prognostic network based on the TCGA‐LIGC cohort consisting of 13 immLysorgs. Each sphere represents the Cox test for a given gene, and the linkage between spheres represents the correlation between genes. immLysorgs, immune lysosome‐related genes. Supporting Information 3 Figure S3: GSVA heat map showing the differences in pathways in the two clusters. (A) Gene set from “c2.cp.kegg.v7.5.1.symbols.gmt.” (B) Gene set from “c2.cp.reactome.v7.5.1.symbols.gmt.” (C) Gene set from “h.all.v7.5.1.symbols.gmt.” Supporting Information 4 Figure S4: Functional enrichment analysis of DEGs between C1 and C2 subgroups. (A) Volcano map of DEGs. (B, D) Analysis of GO‐enriched BP, CC, and MF terms demonstrating the possible role of DEGs. (C, E) Kyoto Encyclopedia of Genes and Genomes (KEGG) pathway enrichment analysis revealing possible pathways. Supporting Information 5 Figure S5: GSEA analysis of differential genes between different immLysoS groups. (A, B) GSEA analysis between high‐ and low‐risk groups using the gene set “c2.cp.reactome.v7.5.1.symbols.gmt.” (C, D) GSEA analysis between high‐ and low‐risk groups using gene set “c5.go.v7.4.symbols.gmt.” Supporting Information 6 Figure S6: Correlation of immLysoS with the tumor immune microenvi [file HUMU-2026-3501996-s001.zip › Figure S1.pdf]

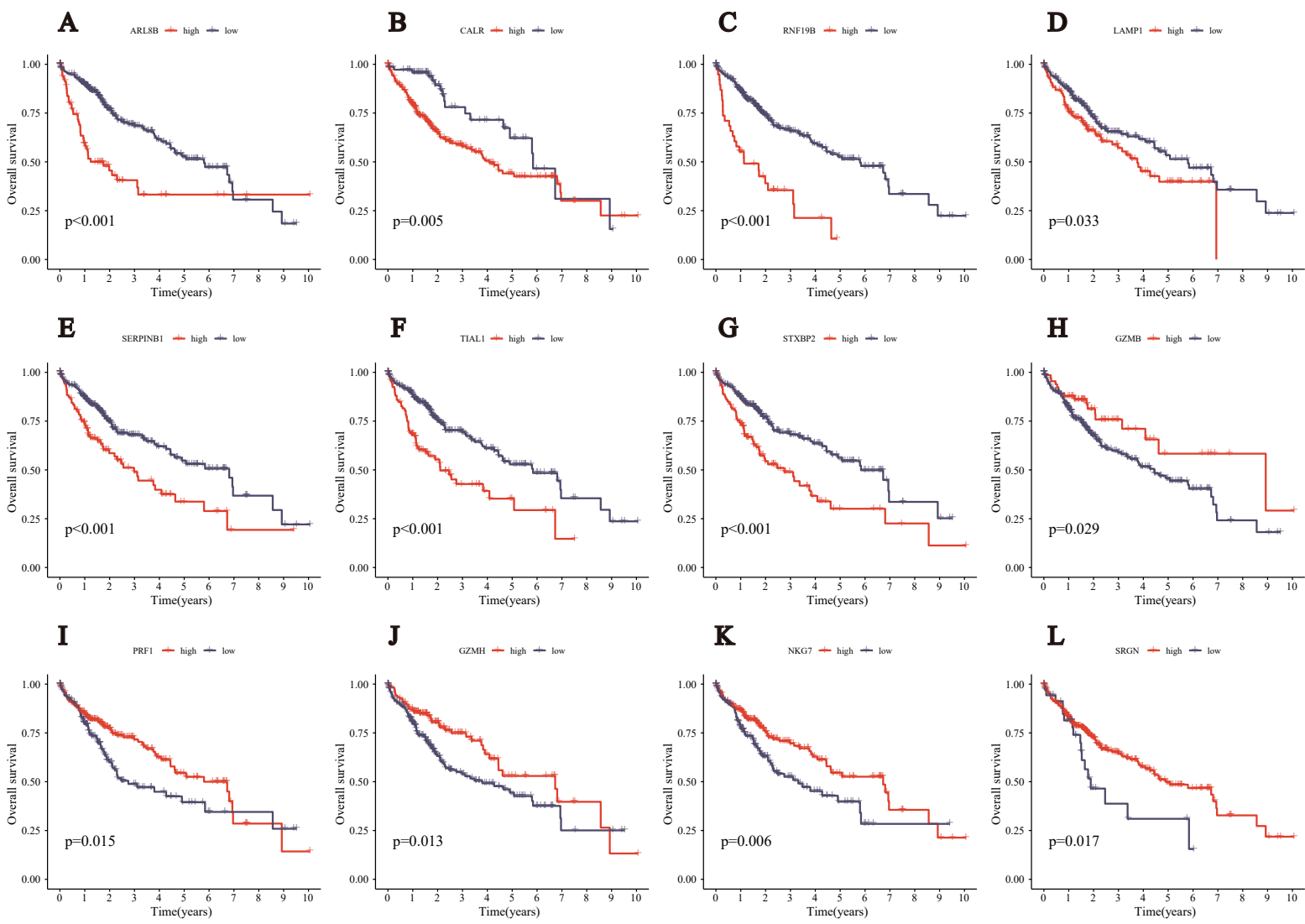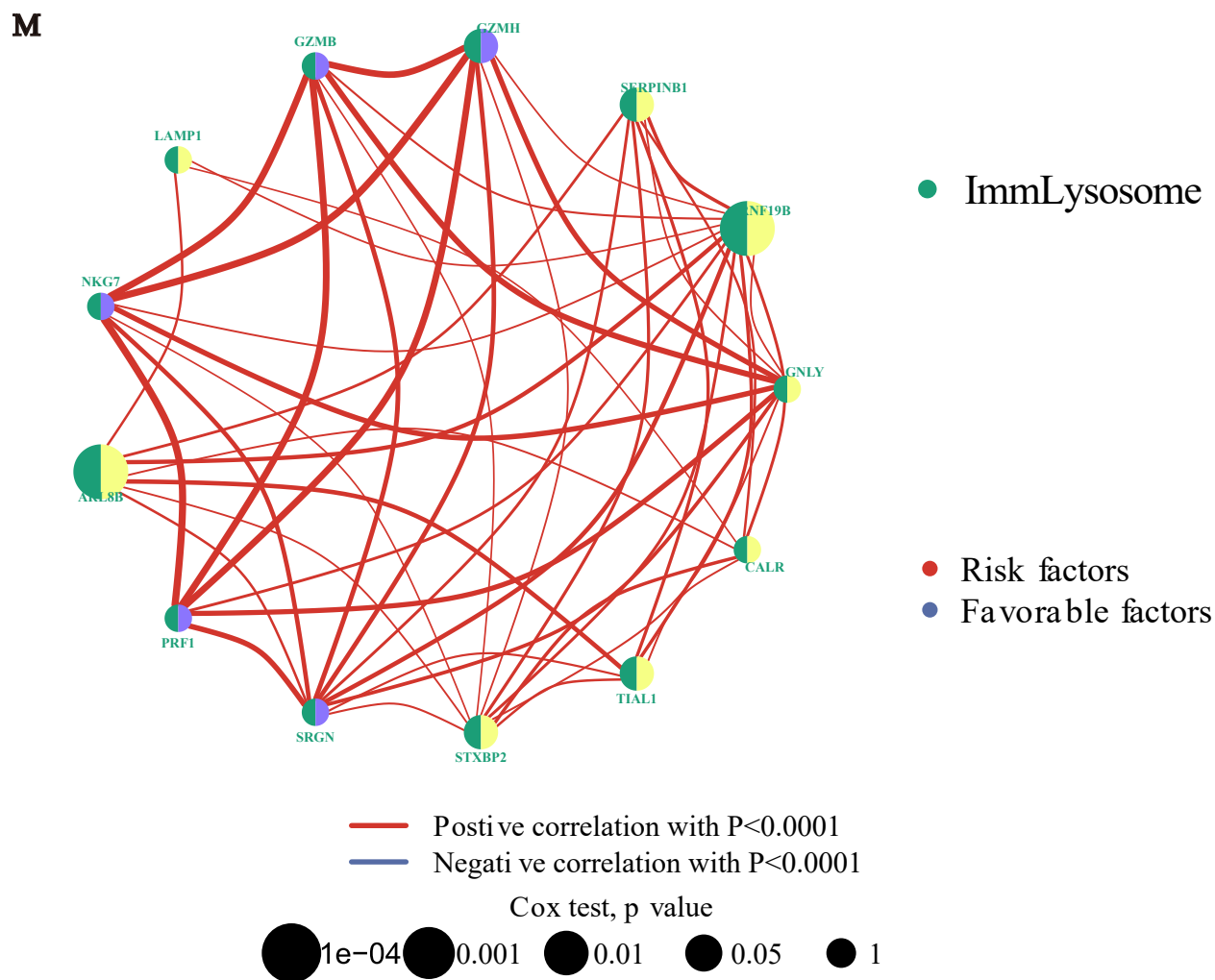

Supplement: Supplementary file 1 — Supporting Information 1 Additional supporting information can be found online in the Supporting Information section. Supporting Information. Figure S1: Expression and genetic alteration of immLysorgs in HCC. (A) The expression of 13 immLysorgs in HCC and normal tissues. (B–D) The mutation frequency and CNV and chromosomal localization of 13 immLysorgs in HCC. ∗ p < 0.05, ∗∗ p < 0.01, and ∗∗∗ p < 0.001; ns, not statistically different; immLysorgs, immune lysosome‐related genes. Supporting Information 2 Figure S2. Prognostic significance of immLysorgs of HCC patients in TCGA. (A–L) Single‐gene K‐M survival analysis based on the TCGA‐LIGC cohort showing that the expression of 12 immLysorgs significantly affects patient prognosis. (M) Correlation prognostic network based on the TCGA‐LIGC cohort consisting of 13 immLysorgs. Each sphere represents the Cox test for a given gene, and the linkage between spheres represents the correlation between genes. immLysorgs, immune lysosome‐related genes. Supporting Information 3 Figure S3: GSVA heat map showing the differences in pathways in the two clusters. (A) Gene set from “c2.cp.kegg.v7.5.1.symbols.gmt.” (B) Gene set from “c2.cp.reactome.v7.5.1.symbols.gmt.” (C) Gene set from “h.all.v7.5.1.symbols.gmt.” Supporting Information 4 Figure S4: Functional enrichment analysis of DEGs between C1 and C2 subgroups. (A) Volcano map of DEGs. (B, D) Analysis of GO‐enriched BP, CC, and MF terms demonstrating the possible role of DEGs. (C, E) Kyoto Encyclopedia of Genes and Genomes (KEGG) pathway enrichment analysis revealing possible pathways. Supporting Information 5 Figure S5: GSEA analysis of differential genes between different immLysoS groups. (A, B) GSEA analysis between high‐ and low‐risk groups using the gene set “c2.cp.reactome.v7.5.1.symbols.gmt.” (C, D) GSEA analysis between high‐ and low‐risk groups using gene set “c5.go.v7.4.symbols.gmt.” Supporting Information 6 Figure S6: Correlation of immLysoS with the tumor immune microenvi [file HUMU-2026-3501996-s001.zip › Figure S2.pdf]

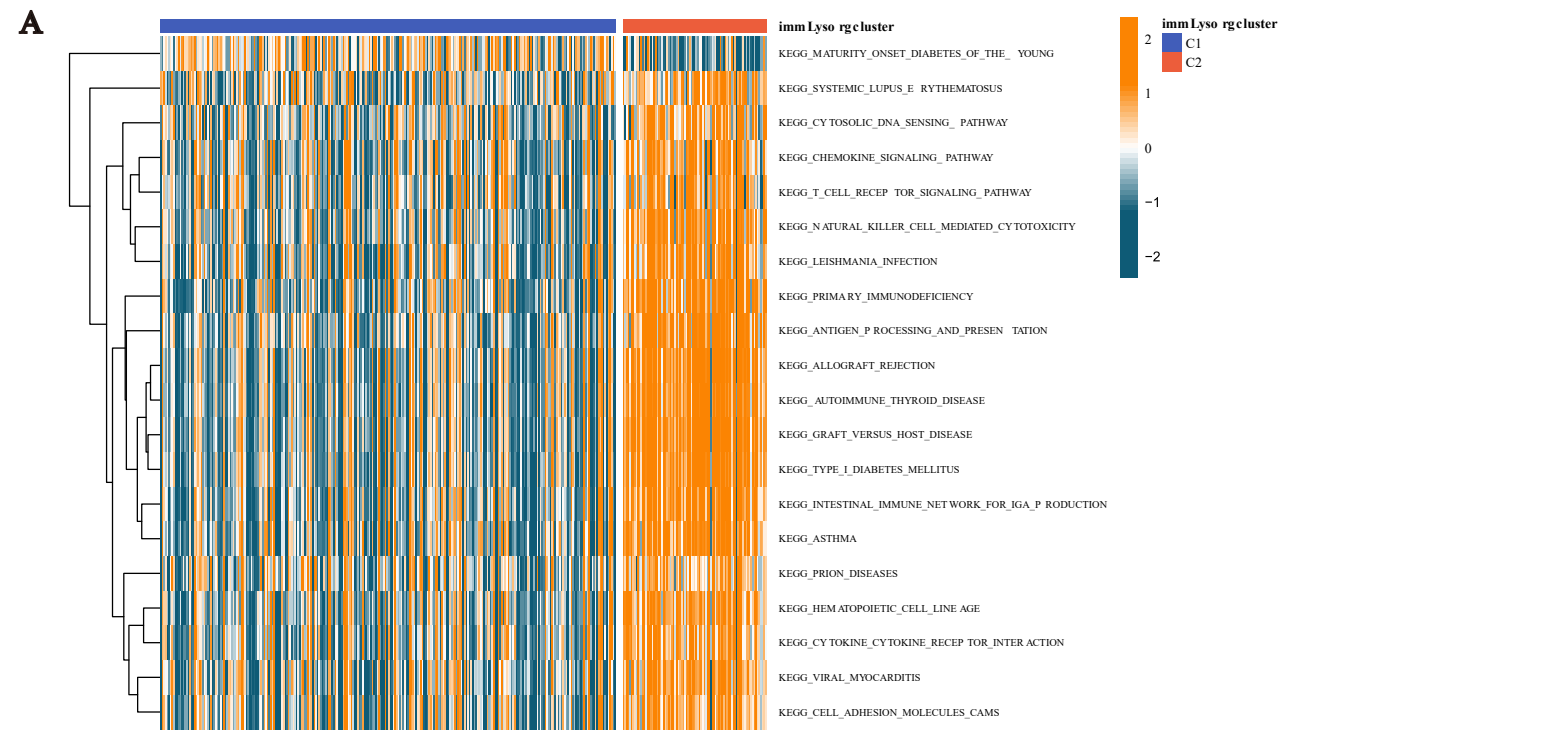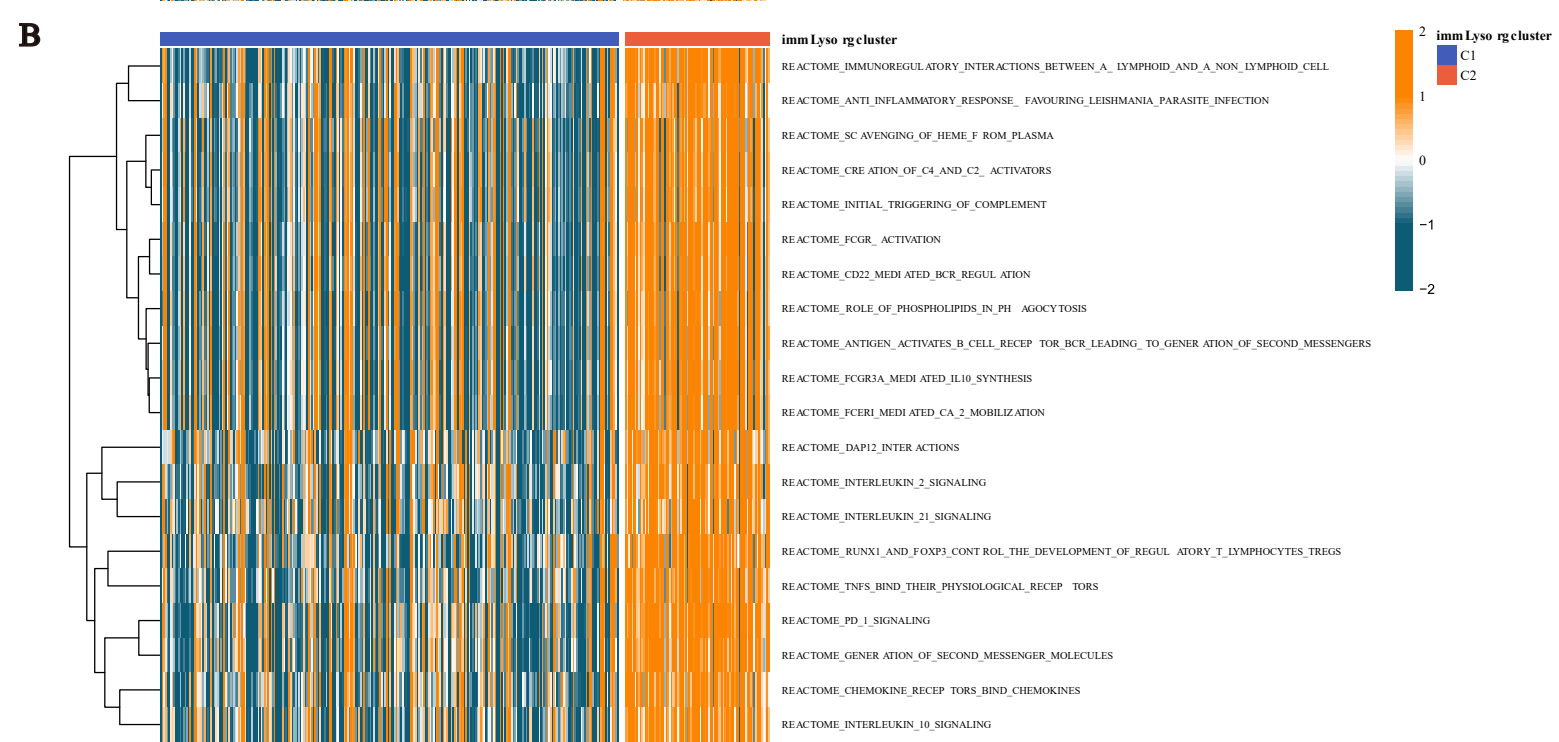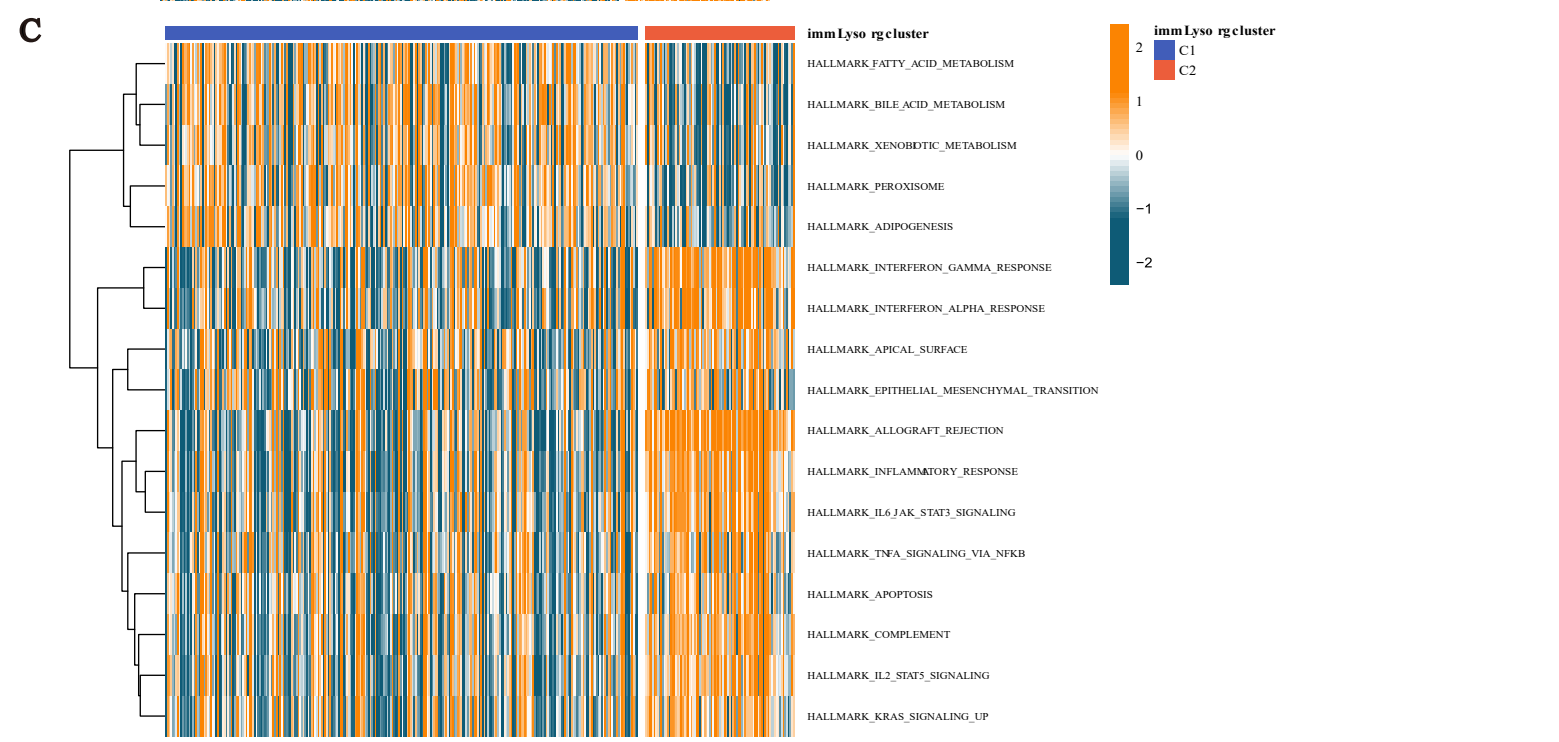

Supplement: Supplementary file 1 — Supporting Information 1 Additional supporting information can be found online in the Supporting Information section. Supporting Information. Figure S1: Expression and genetic alteration of immLysorgs in HCC. (A) The expression of 13 immLysorgs in HCC and normal tissues. (B–D) The mutation frequency and CNV and chromosomal localization of 13 immLysorgs in HCC. ∗ p < 0.05, ∗∗ p < 0.01, and ∗∗∗ p < 0.001; ns, not statistically different; immLysorgs, immune lysosome‐related genes. Supporting Information 2 Figure S2. Prognostic significance of immLysorgs of HCC patients in TCGA. (A–L) Single‐gene K‐M survival analysis based on the TCGA‐LIGC cohort showing that the expression of 12 immLysorgs significantly affects patient prognosis. (M) Correlation prognostic network based on the TCGA‐LIGC cohort consisting of 13 immLysorgs. Each sphere represents the Cox test for a given gene, and the linkage between spheres represents the correlation between genes. immLysorgs, immune lysosome‐related genes. Supporting Information 3 Figure S3: GSVA heat map showing the differences in pathways in the two clusters. (A) Gene set from “c2.cp.kegg.v7.5.1.symbols.gmt.” (B) Gene set from “c2.cp.reactome.v7.5.1.symbols.gmt.” (C) Gene set from “h.all.v7.5.1.symbols.gmt.” Supporting Information 4 Figure S4: Functional enrichment analysis of DEGs between C1 and C2 subgroups. (A) Volcano map of DEGs. (B, D) Analysis of GO‐enriched BP, CC, and MF terms demonstrating the possible role of DEGs. (C, E) Kyoto Encyclopedia of Genes and Genomes (KEGG) pathway enrichment analysis revealing possible pathways. Supporting Information 5 Figure S5: GSEA analysis of differential genes between different immLysoS groups. (A, B) GSEA analysis between high‐ and low‐risk groups using the gene set “c2.cp.reactome.v7.5.1.symbols.gmt.” (C, D) GSEA analysis between high‐ and low‐risk groups using gene set “c5.go.v7.4.symbols.gmt.” Supporting Information 6 Figure S6: Correlation of immLysoS with the tumor immune microenvi [file HUMU-2026-3501996-s001.zip › Figure S3.pdf]

**A**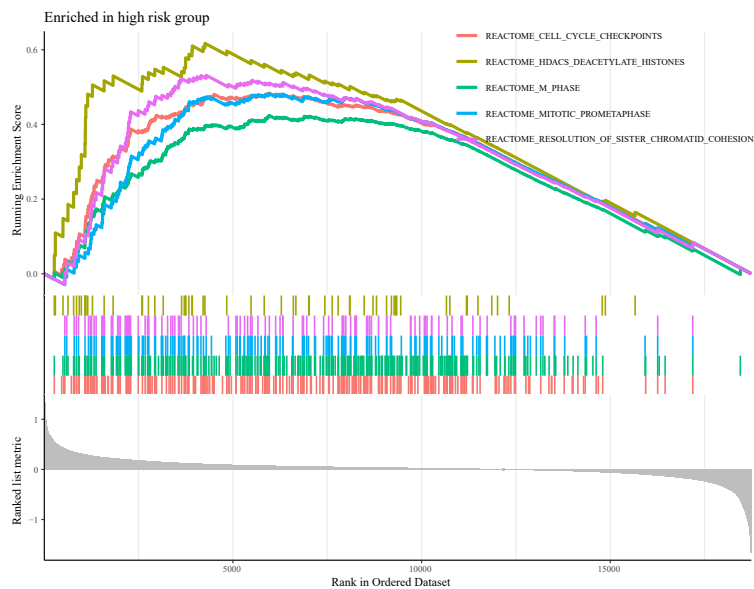**B**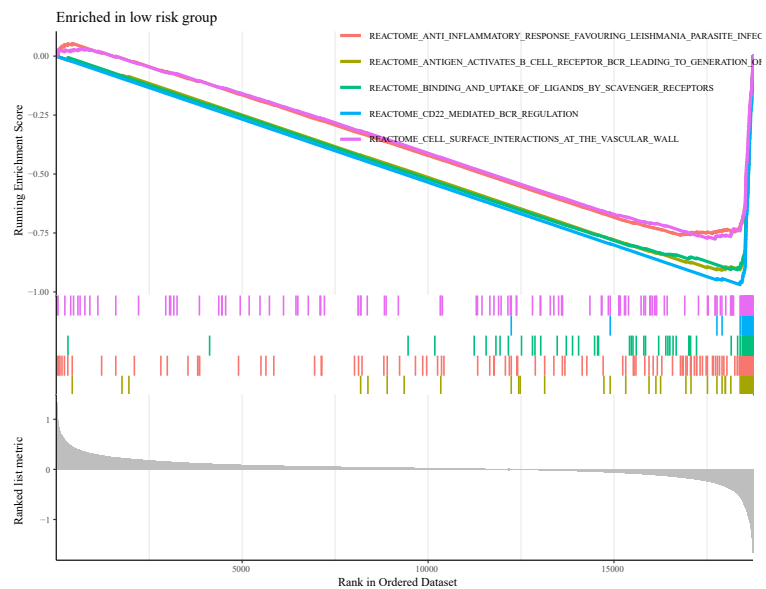**C**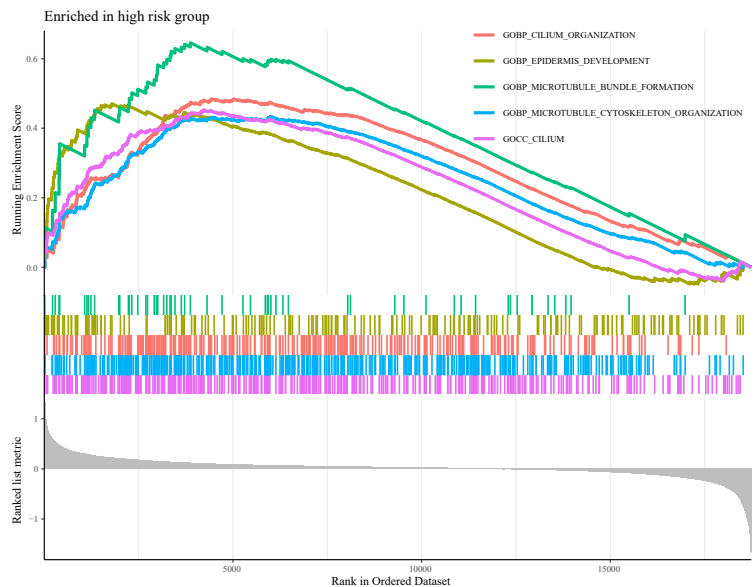**D**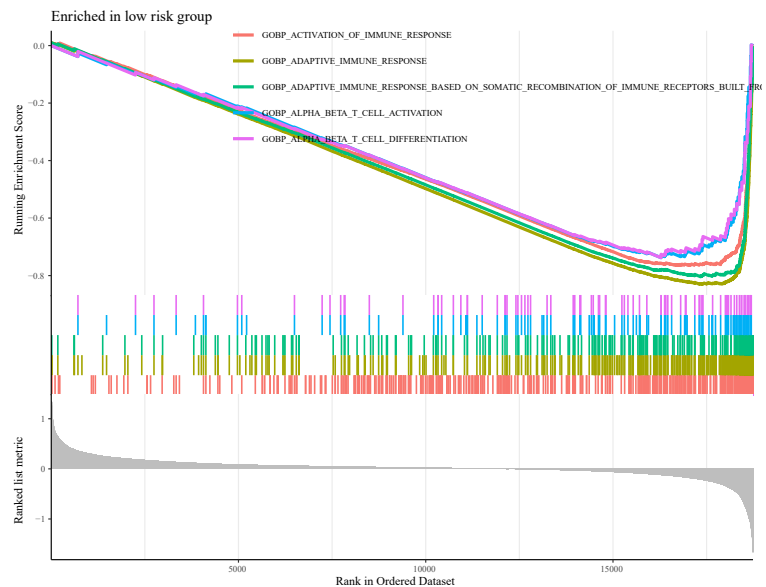

Supplement: Supplementary file 1 — Supporting Information 1 Additional supporting information can be found online in the Supporting Information section. Supporting Information. Figure S1: Expression and genetic alteration of immLysorgs in HCC. (A) The expression of 13 immLysorgs in HCC and normal tissues. (B–D) The mutation frequency and CNV and chromosomal localization of 13 immLysorgs in HCC. ∗ p < 0.05, ∗∗ p < 0.01, and ∗∗∗ p < 0.001; ns, not statistically different; immLysorgs, immune lysosome‐related genes. Supporting Information 2 Figure S2. Prognostic significance of immLysorgs of HCC patients in TCGA. (A–L) Single‐gene K‐M survival analysis based on the TCGA‐LIGC cohort showing that the expression of 12 immLysorgs significantly affects patient prognosis. (M) Correlation prognostic network based on the TCGA‐LIGC cohort consisting of 13 immLysorgs. Each sphere represents the Cox test for a given gene, and the linkage between spheres represents the correlation between genes. immLysorgs, immune lysosome‐related genes. Supporting Information 3 Figure S3: GSVA heat map showing the differences in pathways in the two clusters. (A) Gene set from “c2.cp.kegg.v7.5.1.symbols.gmt.” (B) Gene set from “c2.cp.reactome.v7.5.1.symbols.gmt.” (C) Gene set from “h.all.v7.5.1.symbols.gmt.” Supporting Information 4 Figure S4: Functional enrichment analysis of DEGs between C1 and C2 subgroups. (A) Volcano map of DEGs. (B, D) Analysis of GO‐enriched BP, CC, and MF terms demonstrating the possible role of DEGs. (C, E) Kyoto Encyclopedia of Genes and Genomes (KEGG) pathway enrichment analysis revealing possible pathways. Supporting Information 5 Figure S5: GSEA analysis of differential genes between different immLysoS groups. (A, B) GSEA analysis between high‐ and low‐risk groups using the gene set “c2.cp.reactome.v7.5.1.symbols.gmt.” (C, D) GSEA analysis between high‐ and low‐risk groups using gene set “c5.go.v7.4.symbols.gmt.” Supporting Information 6 Figure S6: Correlation of immLysoS with the tumor immune microenvi [file HUMU-2026-3501996-s001.zip › Figure S5.pdf]

A

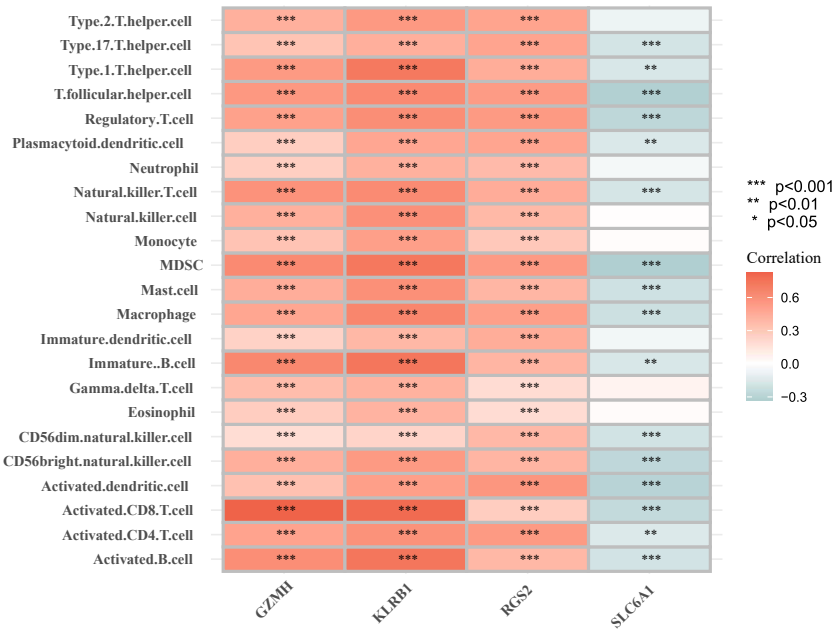

B

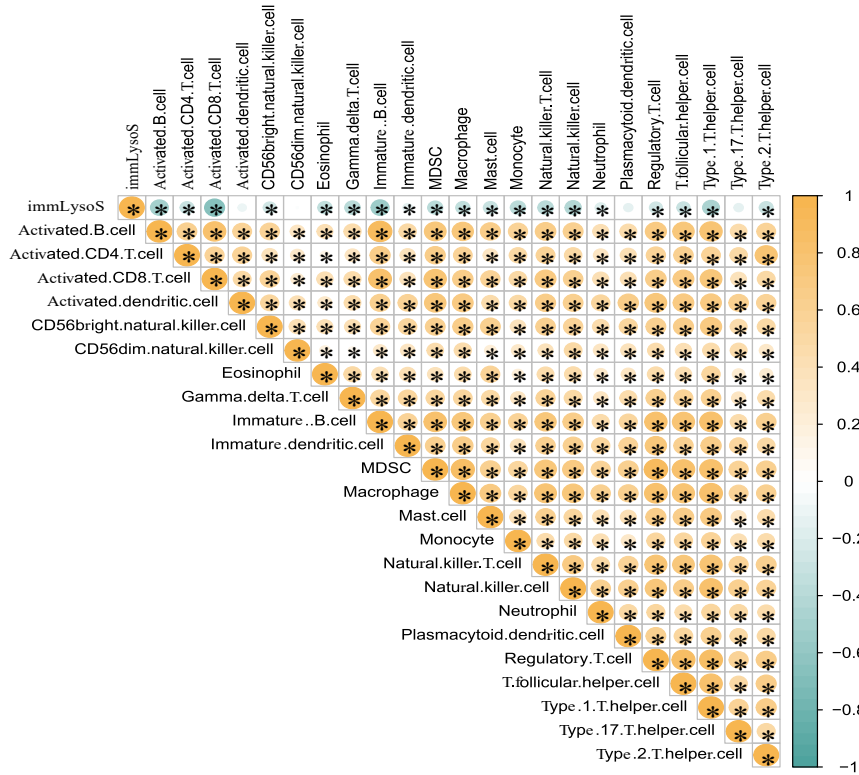

F

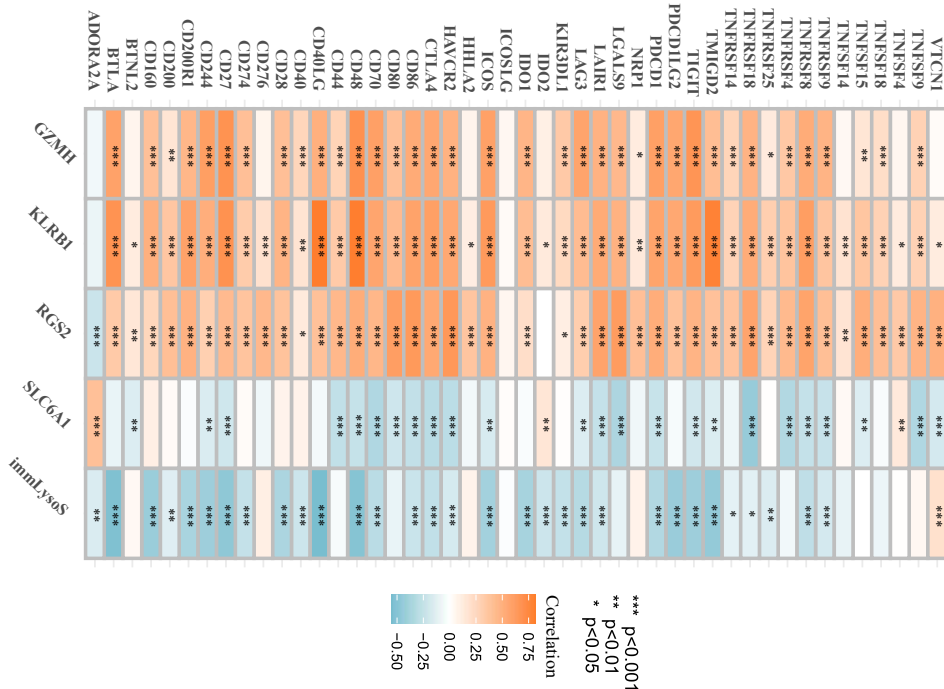

Supplement: Supplementary file 1 — Supporting Information 1 Additional supporting information can be found online in the Supporting Information section. Supporting Information. Figure S1: Expression and genetic alteration of immLysorgs in HCC. (A) The expression of 13 immLysorgs in HCC and normal tissues. (B–D) The mutation frequency and CNV and chromosomal localization of 13 immLysorgs in HCC. ∗ p < 0.05, ∗∗ p < 0.01, and ∗∗∗ p < 0.001; ns, not statistically different; immLysorgs, immune lysosome‐related genes. Supporting Information 2 Figure S2. Prognostic significance of immLysorgs of HCC patients in TCGA. (A–L) Single‐gene K‐M survival analysis based on the TCGA‐LIGC cohort showing that the expression of 12 immLysorgs significantly affects patient prognosis. (M) Correlation prognostic network based on the TCGA‐LIGC cohort consisting of 13 immLysorgs. Each sphere represents the Cox test for a given gene, and the linkage between spheres represents the correlation between genes. immLysorgs, immune lysosome‐related genes. Supporting Information 3 Figure S3: GSVA heat map showing the differences in pathways in the two clusters. (A) Gene set from “c2.cp.kegg.v7.5.1.symbols.gmt.” (B) Gene set from “c2.cp.reactome.v7.5.1.symbols.gmt.” (C) Gene set from “h.all.v7.5.1.symbols.gmt.” Supporting Information 4 Figure S4: Functional enrichment analysis of DEGs between C1 and C2 subgroups. (A) Volcano map of DEGs. (B, D) Analysis of GO‐enriched BP, CC, and MF terms demonstrating the possible role of DEGs. (C, E) Kyoto Encyclopedia of Genes and Genomes (KEGG) pathway enrichment analysis revealing possible pathways. Supporting Information 5 Figure S5: GSEA analysis of differential genes between different immLysoS groups. (A, B) GSEA analysis between high‐ and low‐risk groups using the gene set “c2.cp.reactome.v7.5.1.symbols.gmt.” (C, D) GSEA analysis between high‐ and low‐risk groups using gene set “c5.go.v7.4.symbols.gmt.” Supporting Information 6 Figure S6: Correlation of immLysoS with the tumor immune microenvi [file HUMU-2026-3501996-s001.zip › Figure S6.pdf]

**A**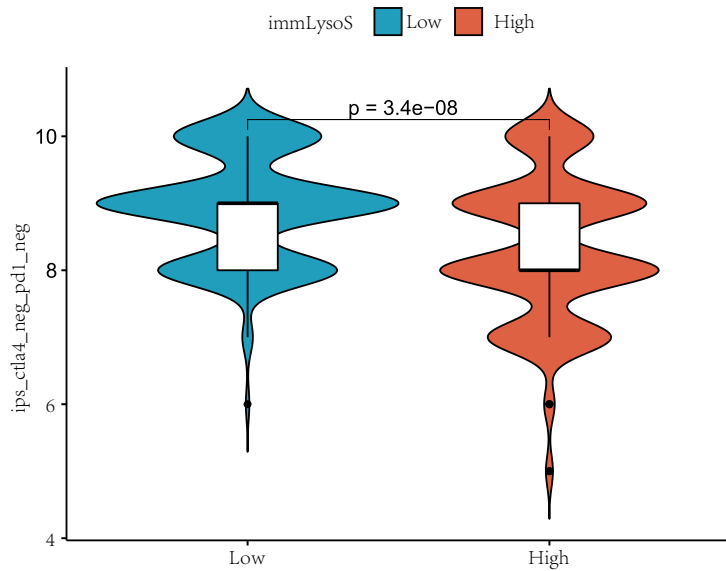**B**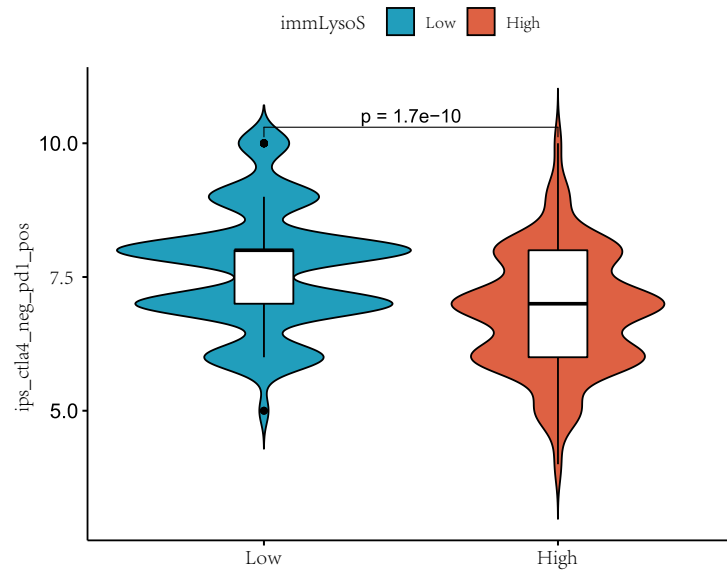**C**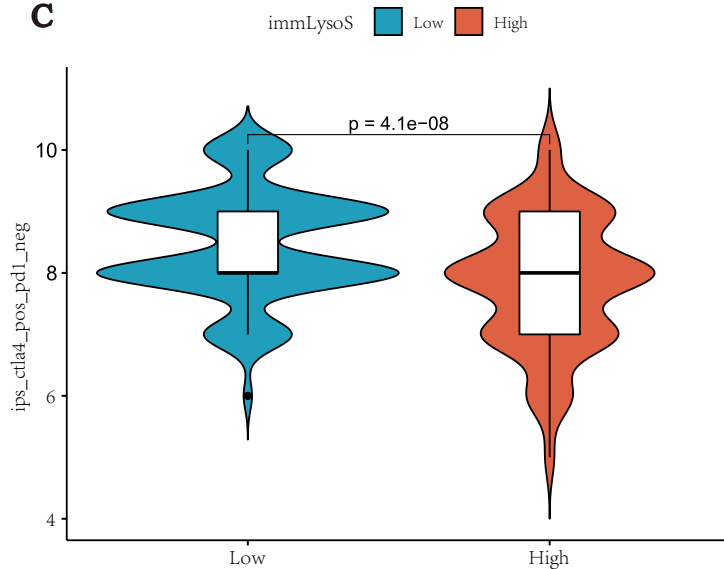**D**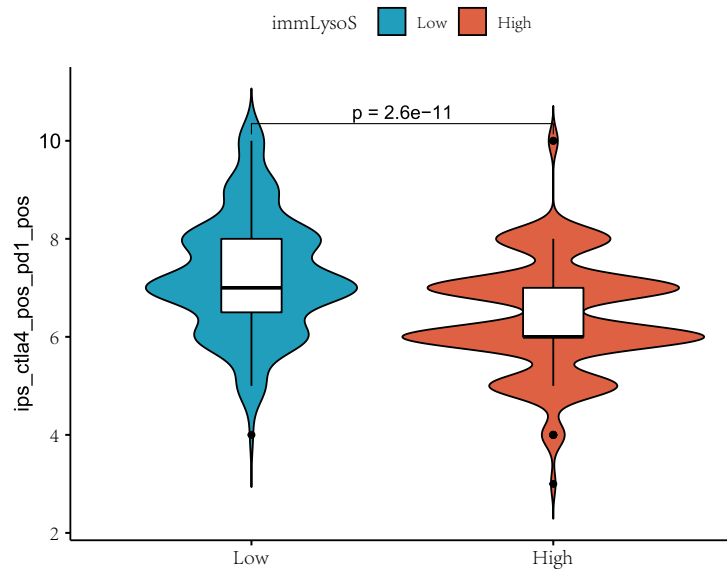**E**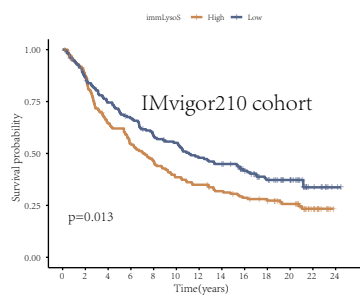**F**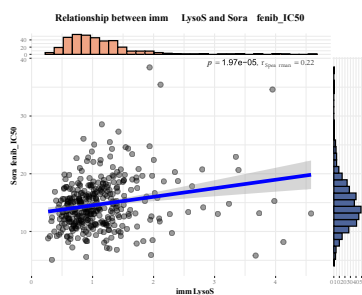**G**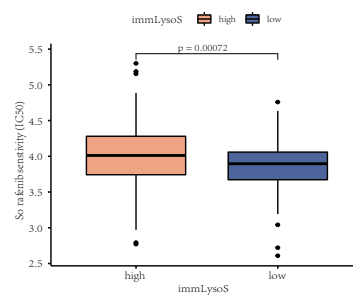**H**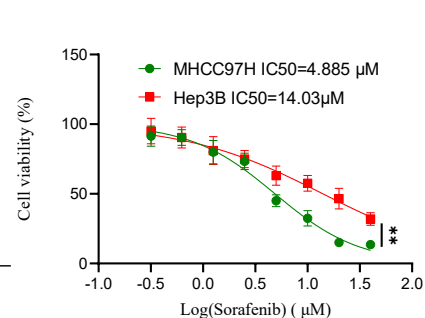

Supplement: Supplementary file 1 — Supporting Information 1 Additional supporting information can be found online in the Supporting Information section. Supporting Information. Figure S1: Expression and genetic alteration of immLysorgs in HCC. (A) The expression of 13 immLysorgs in HCC and normal tissues. (B–D) The mutation frequency and CNV and chromosomal localization of 13 immLysorgs in HCC. ∗ p < 0.05, ∗∗ p < 0.01, and ∗∗∗ p < 0.001; ns, not statistically different; immLysorgs, immune lysosome‐related genes. Supporting Information 2 Figure S2. Prognostic significance of immLysorgs of HCC patients in TCGA. (A–L) Single‐gene K‐M survival analysis based on the TCGA‐LIGC cohort showing that the expression of 12 immLysorgs significantly affects patient prognosis. (M) Correlation prognostic network based on the TCGA‐LIGC cohort consisting of 13 immLysorgs. Each sphere represents the Cox test for a given gene, and the linkage between spheres represents the correlation between genes. immLysorgs, immune lysosome‐related genes. Supporting Information 3 Figure S3: GSVA heat map showing the differences in pathways in the two clusters. (A) Gene set from “c2.cp.kegg.v7.5.1.symbols.gmt.” (B) Gene set from “c2.cp.reactome.v7.5.1.symbols.gmt.” (C) Gene set from “h.all.v7.5.1.symbols.gmt.” Supporting Information 4 Figure S4: Functional enrichment analysis of DEGs between C1 and C2 subgroups. (A) Volcano map of DEGs. (B, D) Analysis of GO‐enriched BP, CC, and MF terms demonstrating the possible role of DEGs. (C, E) Kyoto Encyclopedia of Genes and Genomes (KEGG) pathway enrichment analysis revealing possible pathways. Supporting Information 5 Figure S5: GSEA analysis of differential genes between different immLysoS groups. (A, B) GSEA analysis between high‐ and low‐risk groups using the gene set “c2.cp.reactome.v7.5.1.symbols.gmt.” (C, D) GSEA analysis between high‐ and low‐risk groups using gene set “c5.go.v7.4.symbols.gmt.” Supporting Information 6 Figure S6: Correlation of immLysoS with the tumor immune microenvi [file HUMU-2026-3501996-s001.zip › Figure S7.pdf]

A

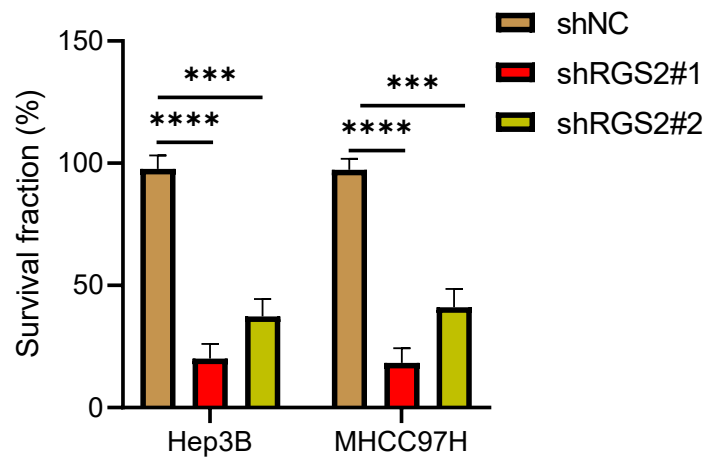

B

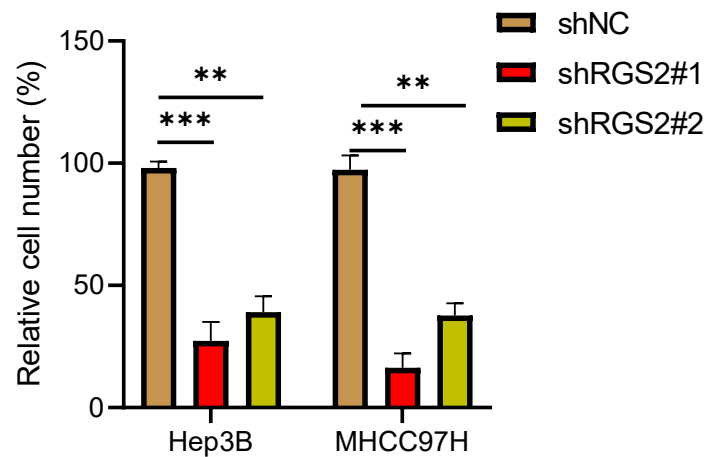

C

shNC

shRGS#1

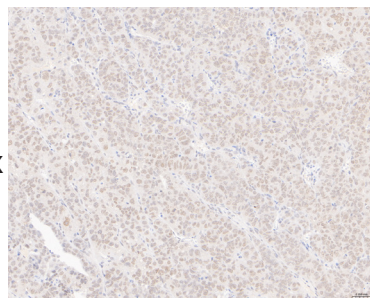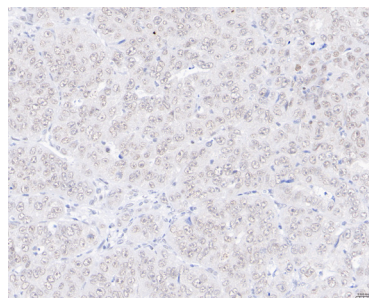

20x

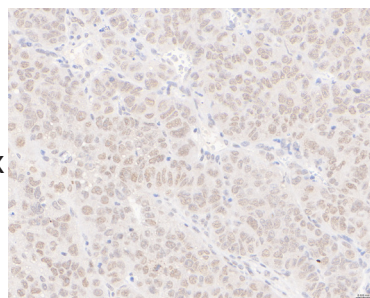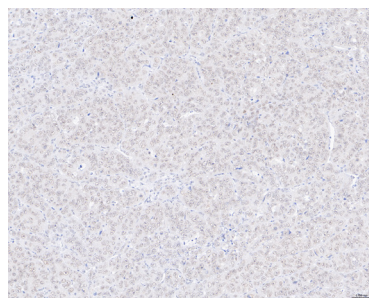

40x

RGS2

D

shNC

shRGS#1

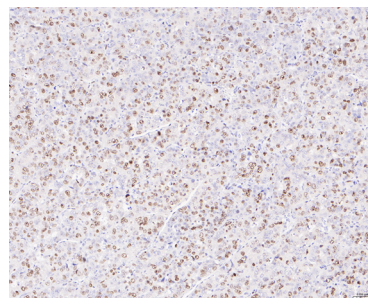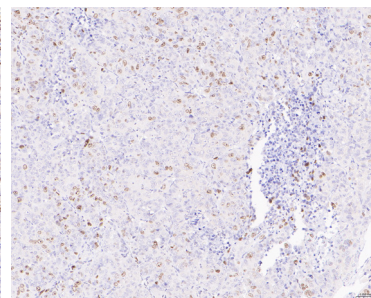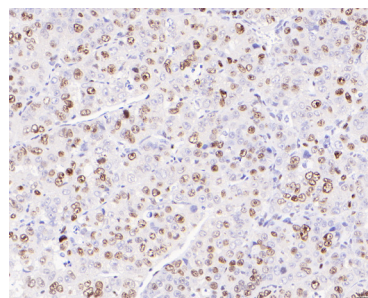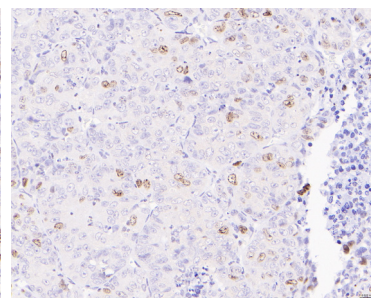

Ki67

Supplement: Supplementary file 1 — Supporting Information 1 Additional supporting information can be found online in the Supporting Information section. Supporting Information. Figure S1: Expression and genetic alteration of immLysorgs in HCC. (A) The expression of 13 immLysorgs in HCC and normal tissues. (B–D) The mutation frequency and CNV and chromosomal localization of 13 immLysorgs in HCC. ∗ p < 0.05, ∗∗ p < 0.01, and ∗∗∗ p < 0.001; ns, not statistically different; immLysorgs, immune lysosome‐related genes. Supporting Information 2 Figure S2. Prognostic significance of immLysorgs of HCC patients in TCGA. (A–L) Single‐gene K‐M survival analysis based on the TCGA‐LIGC cohort showing that the expression of 12 immLysorgs significantly affects patient prognosis. (M) Correlation prognostic network based on the TCGA‐LIGC cohort consisting of 13 immLysorgs. Each sphere represents the Cox test for a given gene, and the linkage between spheres represents the correlation between genes. immLysorgs, immune lysosome‐related genes. Supporting Information 3 Figure S3: GSVA heat map showing the differences in pathways in the two clusters. (A) Gene set from “c2.cp.kegg.v7.5.1.symbols.gmt.” (B) Gene set from “c2.cp.reactome.v7.5.1.symbols.gmt.” (C) Gene set from “h.all.v7.5.1.symbols.gmt.” Supporting Information 4 Figure S4: Functional enrichment analysis of DEGs between C1 and C2 subgroups. (A) Volcano map of DEGs. (B, D) Analysis of GO‐enriched BP, CC, and MF terms demonstrating the possible role of DEGs. (C, E) Kyoto Encyclopedia of Genes and Genomes (KEGG) pathway enrichment analysis revealing possible pathways. Supporting Information 5 Figure S5: GSEA analysis of differential genes between different immLysoS groups. (A, B) GSEA analysis between high‐ and low‐risk groups using the gene set “c2.cp.reactome.v7.5.1.symbols.gmt.” (C, D) GSEA analysis between high‐ and low‐risk groups using gene set “c5.go.v7.4.symbols.gmt.” Supporting Information 6 Figure S6: Correlation of immLysoS with the tumor immune microenvi [file HUMU-2026-3501996-s001.zip › Figure S8.pdf]
