# Supplementary material for: Secretory Lysosome‐Related Gene Signature Defines the Immune Microenvironment and Identifies RGS2 as a Prometastatic Factor in Hepatocellular Carcinoma
Source: Hum Mutat. 2026 May 16;2026:3501996. doi: 10.1155/humu/3501996 (PMC13179717; doi:10.1155/humu/3501996)
Supplement: Supplementary file 1 — Supporting Information 1 Additional supporting information can be found online in the Supporting Information section. Supporting Information. Figure S1: Expression and genetic alteration of immLysorgs in HCC. (A) The expression of 13 immLysorgs in HCC and normal tissues. (B–D) The mutation frequency and CNV and chromosomal localization of 13 immLysorgs in HCC. ∗ p < 0.05, ∗∗ p < 0.01, and ∗∗∗ p < 0.001; ns, not statistically different; immLysorgs, immune lysosome‐related genes. Supporting Information 2 Figure S2. Prognostic significance of immLysorgs of HCC patients in TCGA. (A–L) Single‐gene K‐M survival analysis based on the TCGA‐LIGC cohort showing that the expression of 12 immLysorgs significantly affects patient prognosis. (M) Correlation prognostic network based on the TCGA‐LIGC cohort consisting of 13 immLysorgs. Each sphere represents the Cox test for a given gene, and the linkage between spheres represents the correlation between genes. immLysorgs, immune lysosome‐related genes. Supporting Information 3 Figure S3: GSVA heat map showing the differences in pathways in the two clusters. (A) Gene set from “c2.cp.kegg.v7.5.1.symbols.gmt.” (B) Gene set from “c2.cp.reactome.v7.5.1.symbols.gmt.” (C) Gene set from “h.all.v7.5.1.symbols.gmt.” Supporting Information 4 Figure S4: Functional enrichment analysis of DEGs between C1 and C2 subgroups. (A) Volcano map of DEGs. (B, D) Analysis of GO‐enriched BP, CC, and MF terms demonstrating the possible role of DEGs. (C, E) Kyoto Encyclopedia of Genes and Genomes (KEGG) pathway enrichment analysis revealing possible pathways. Supporting Information 5 Figure S5: GSEA analysis of differential genes between different immLysoS groups. (A, B) GSEA analysis between high‐ and low‐risk groups using the gene set “c2.cp.reactome.v7.5.1.symbols.gmt.” (C, D) GSEA analysis between high‐ and low‐risk groups using gene set “c5.go.v7.4.symbols.gmt.” Supporting Information 6 Figure S6: Correlation of immLysoS with the tumor immune microenvi [file Figure S4.pdf › Figure S4.pdf]

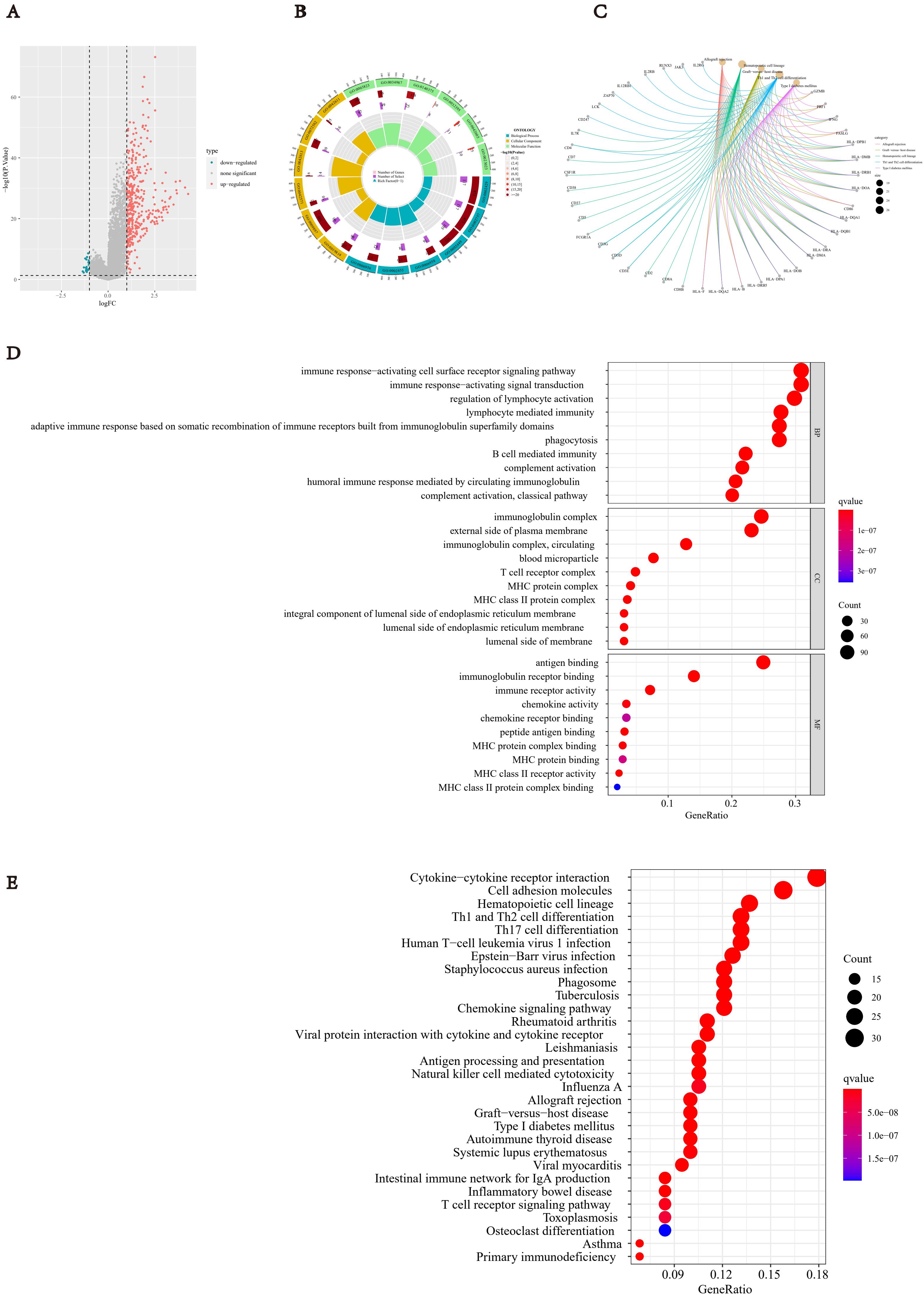
A
B
C
D
E
type
down−regulated
none significant
up−regulated
−log10(P.Value)
logFC
ONTOLOGY
Biological Process
Cellular Component
Molecular Function
immune response−activating cell surface receptor signaling pathway
immune response−activating signal transduction
regulation of lymphocyte activation
lymphocyte mediated immunity
adaptive immune response based on somatic recombination of immune receptors built from immunoglobulin superfamily domains
phagocytosis
B cell mediated immunity
complement activation
humoral immune response mediated by circulating immunoglobulin
complement activation, classical pathway
immunoglobulin complex
external side of plasma membrane
immunoglobulin complex, circulating
blood microparticle
T cell receptor complex
MHC protein complex
MHC class II protein complex
integral component of lumenal side of endoplasmic reticulum membrane
lumenal side of endoplasmic reticulum membrane
lumenal side of membrane
antigen binding
immunoglobulin receptor binding
immune receptor activity
chemokine activity
chemokine receptor binding
peptide antigen binding
MHC protein complex binding
MHC protein binding
MHC class II receptor activity
MHC class II protein complex binding
BP
CC
MF
GeneRatio
qvalue
Count
Cytokine−cytokine receptor interaction
Cell adhesion molecules
Hematopoietic cell lineage
Th1 and Th2 cell differentiation
Th17 cell differentiation
Human T−cell leukemia virus 1 infection
Epstein−Barr virus infection
Staphylococcus aureus infection
Phagosome
Tuberculosis
Chemokine signaling pathway
Rheumatoid arthritis
Viral protein interaction with cytokine and cytokine receptor
Leishmaniasis
Antigen processing and presentation
Natural killer cell mediated cytotoxicity
Influenza A
Allograft rejection
Graft−versus−host disease
Type I diabetes mellitus
Autoimmune thyroid disease
Systemic lupus erythematosus
Viral myocarditis
Intestinal immune network for IgA production
Inflammatory bowel disease
T cell receptor signaling pathway
Toxoplasmosis
Osteoclast differentiation
Asthma
Primary immunodeficiency
GeneRatio
Count
qvalue
